# Psychological Predictors of Self-reported COVID-19 Outcomes: Results From a Prospective Cohort Study

**DOI:** 10.1093/abm/kaab106

**Published:** 2022-01-03

**Authors:** Kieran Ayling, Ru Jia, Carol Coupland, Trudie Chalder, Adam Massey, Elizabeth Broadbent, Kavita Vedhara

**Affiliations:** 1 Centre for Academic Primary Care, University of Nottingham, University Park, Nottingham, UK; 2 Department of Psychological Medicine, Institute of Psychiatry, Psychology & Neuroscience, King’s College London, London, UK; 3 Department of Psychological Medicine, University of Auckland, Auckland, New Zealand

**Keywords:** COVID-19, Mental health, Depression, Anxiety, Psychoneuroimmunology, Infection

## Abstract

**Background:**

Previous research has shown that psychological factors, such as stress and social support, are associated with greater susceptibility to viral respiratory illnesses and more severe symptoms. During the COVID-19 pandemic there has been a well-documented deterioration in psychological well-being and increased social isolation. This raises questions as to whether those experiencing psychological adversity during the pandemic are more at risk of contracting and/or experiencing COVID-19 symptoms.

**Purpose:**

To examine the relationship between psychological factors and the risk of COVID-19 self-reported infection and the symptomatic experience of SARS-CoV-2 (indicated by the number and severity of symptoms).

**Methods:**

As part of a longitudinal prospective observational cohort study, 1,087 adults completed validated measures of psychological well-being during April 2020 and self-reported incidence of COVID-19 infection and symptom experience across the pandemic through to December 2020. Regression models were used to explore these relationships controlling for demographic and occupational factors.

**Results:**

Greater psychological distress during the early phase of the pandemic was significantly associated with subsequent self-reported SARS-CoV-2 infection as well as the experience of a greater number and more severe symptoms.

**Conclusions:**

COVID-19 infection and symptoms may be more common among those experiencing elevated psychological distress. Further research to elucidate the mechanisms underlying these associations is needed.

## Teaser

Emotional distress during the pandemic was associated with an increased risk of becoming infected with COVID-19 as well as experiencing a greater number and more severe symptoms.

## Introduction

The COVID-19 (Coronavirus, 2019) pandemic has resulted in unprecedented disruption to the fabric of societies, health services, and economies. The multitude of challenges unleashed by the pandemic has necessarily affected psychological well-being too. Increases in a range of mental health difficulties (e.g., anxiety and depression) and risk factors associated with poorer mental health (e.g., loneliness) have been reported in many cohorts and across many countries [1–4]. In view of the now well-established associations between adverse emotional experiences and physical health [5–7], these observations raise important questions about the role of these psychological outcomes as not only consequences of the COVID-19 pandemic, but also risk factors for the disease itself. We report here findings from a prospective cohort study in which we examined the relationship between indices of psychological functioning reported during the pandemic (reported on two occasions: April 2020 and July–September 2020) and self-reports of COVID-19 infection and symptom severity (reported in November–December 2020).

Previous research has shown that—in addition to a host of demographic and clinical factors (such as age, health status, and medication)—both vulnerability to viral diseases and the likelihood of infection becoming symptomatic are associated with a range of psychological and social factors [8, 9]. Three main areas of enquiry have done much to elucidate this relationship. First, studies examining the role of biopsychosocial factors in immune responses to viral vaccinations (e.g., influenza vaccines) [10]; second, the reactivation of latent viral infections that suddenly become active and/or symptomatic despite lying dormant sometimes for years (e.g., Epstein–Barr virus [11]) and third, viral challenge studies [8]. Of these, research related to viral challenge is of greatest relevance to the present work.

The viral challenge paradigm typically involves quarantining healthy volunteers for several days during which they are exposed to one or more respiratory viruses and then followed up for evidence of infection and/or the presence of symptomatic illness. The methodological elegance of this approach lies in the fact that these studies typically control for previous exposure (by measuring antibody levels at baseline) and viral exposure (i.e., the “dose” of virus to which individuals are exposed) is controlled. One of the first, and perhaps most well-known studies in this area provided evidence of a dose response relationship between a composite measure of psychological stress (stressful life events, negative affect, and perceived stress) and the likelihood of both infection and the severity of subsequent illness (as judged by a physician) [12]. These results not only showed that increases in stress predicted an increased risk of developing a respiratory illness; but also that these effects occurred across a range of different viruses (rhinovirus type 2, 9, 14, respiratory syncytial virus, and coronavirus type 229E) and that the relationship between stress and infection was much stronger than the relationship between stress and illness. This is perhaps unsurprising as we would expect the immune system, in otherwise healthy individuals, to contain and eradicate most infections before they result in symptoms of disease.

Since this ground-breaking work, the viral challenge paradigm has done much to elucidate the connections between psychological and social factors and viral infection/illness. For example, in terms of the common cold at least, the greatest risk of illness occurs in individuals contending with chronic stressors (of 1 month or longer in duration) and where the sources of stress are interpersonal or employment related [13]. There is also evidence that risk of disease is related to both objective and subjective measures of socioeconomic status: with greater risk evident in people with low socioeconomic status in childhood; individuals defined as underemployed or unemployed, and also in people who self-report a lower “perceived social status” [14, 15]. In comparison, the experience of positive emotions and social support confer protection against viral illness. For example, the experience of positive emotions is associated with a lower risk of illness: an effect independent of negative emotions [16]. Similarly, several indices of social support (social integration, perceived social support, and hugging) are also related to reduced vulnerability to viral diseases [17–19].

The evidence above, combined with the knowledge that susceptibility to COVID-19, the symptom experience and progression of the disease are not uniform [20–24], permits us to hypothesize that some of the variability in vulnerability to and outcomes associated with COVID-19 infection may be determined by psychological parameters [25].

In the present work, we sought to examine whether these aforementioned relationships between psychological indices and risk of viral infection and symptomatic illness were evident in the context of COVID-19. We focused primarily on psychological indices which have previously been shown to be related to outcomes in viral diseases [24] including depression, anxiety, stress, positive mood, and loneliness as well as fear of getting COVID-19. We explored whether, after controlling for demographic factors known to be associated with an increased risk of COVID-19 infection, psychological variables predicted self-reported COVID-19 infection and the symptom experience.

## Methods

### Ethics, Recruitment, and Eligibility

Ethical approval was granted from the University of Nottingham Faculty of Medicine and Health Sciences (ref: 506-2003) and the NHS Health Research Authority (ref: 20/HRA/1858). The study was launched on 3/4/20 with participants recruited in the community through a social and mainstream media campaign involving, but not limited to, Facebook and Twitter. In addition, HRA regulatory approval enabled us to approach NHS organizations and request they advertise the research through their routine communications. Recruitment continued until 30/4/20. All media directed potential participants to the study website (www.covidstressstudy.com) through which they accessed the information sheet, consent form, and online survey.

Eligibility criteria specified that participants should be: aged 18 and over; able to give informed consent; able to read English; residing in the UK at the time of completing the survey and able to provide a sample of hair at least 1 cm long. The latter was collected for the determination of the stress biomarker cortisol which will be the subject of future manuscripts.

### Patient and Public Involvement

We convened a virtual patient and public involvement group to support this research the aims of which were to advise on the development of the survey, the participant information sheet, and optimizing recruitment and retention. Individuals participated via MS Teams in one-to-one or group discussions. These discussions informed the length and structure of the survey, language of the information sheet, and strategies for recruiting via media and social media. The views of this group were instrumental in achieving our large sample size. This group also advised on providing regular feedback to participants on study findings through the study website and between each wave of data collection.

### Sample Size

The present cohort was primarily recruited to track the psychological and physical effects of the COVID-19 pandemic on the UK population. As such, we did not place an upper limit on participant numbers to enable us to obtain as precise estimates of population values and associations as possible, and to be able to examine these in smaller subgroups where applicable. At conception, the minimum sample size for the cohort study was calculated to allow for pre–post comparisons on psychological variables collected during waves 1 and 2, with a minimum of 787 participants needed to detect a small effect size (dz = 0.1) with 80% power.

### Procedures

Consenting participants completed an online survey implemented through JISC Online Survey (https://www.onlinesurveys.ac.uk/). Three waves of data collection took place capturing specific periods during the UK pandemic in 2020 [26]. The first occurred between 3/4/20 and 30/4/20 (coinciding with the first national lockdown), the second occurred between 1/7/20 and 21/9/20 (coinciding with an easing of restrictions) and the final one occurred between 11/11/20 and 31/12/20 (coinciding with the second national lockdown). 3,097 participants responded at wave 1, with 1,385 of these responding at wave 2, 1,087 responding at wave 3, and 879 completing all three waves.

In all waves, the survey included measures designed to capture psychological factors which have previously been shown to be related to outcomes in viral diseases (stress, anxiety, depression, loneliness, and positive mood) and factors which we considered to be particularly relevant to the COVID-19 pandemic (worry regarding contracting COVID-19). Depression was measured using the 9-item Patient Health Questionnaire (PHQ-9, α = 0.87), PHQ-9 scores range from 0 to 27 with higher scores indicating worse levels of depression severity. Anxiety was measured using the 7-item Generalized Anxiety Disorder Scale (GAD-7, α = 0.91), GAD-7 scores range from 0 to 21 with higher scores indicating worse anxiety levels. Stress was measured using the 4-item Perceived Stress Scale (PSS-4, α = 0.76), PSS-4 scores range from 0 to 16 with higher scores indicating higher levels of stress. Positive mood was measured using the six positively valanced items from the Scale of Positive and Negative Experience (SPANE, α = 0.94) [24]. Total scores of positive mood range from 6 to 30 with higher scores indicating greater positive mood. Worry regarding contracting COVID-19 and perceived loneliness were measured using single items which are described in Supplementary Appendix S1.

We also measured demographic and occupational factors at baseline known to be associated with an increased risk of, and exposure to, COVID-19 infection. These included age, gender, ethnicity, keyworker status, and COVID-19 risk status. These items are also described in Supplementary Appendix S1.

At wave 3 we captured our self-reported COVID-19 outcomes (for wording see Supplementary Appendix S1). For COVID-19 infection, we captured these data in two ways. First, we asked people to report if they had ever been tested for COVID-19 and their test result. In addition, we asked people to report if they believed they had ever had COVID-19, regardless of whether they had been tested. We considered this appropriate because structural, economic, and behavioral barriers have, and continue to exist in relation to both access to, and uptake of, COVID-19 testing. In terms of access, testing capacity was severely limited in the UK for several months during the pandemic. Thus, the public health advice was to self-isolate if you had symptoms rather than seek testing to verify a diagnosis [26]. As access to testing improved psychological, social, and economic barriers to getting tested became evident (e.g., lack of trust, loss of income, risks of acquiring infection) all of which have combined to reduce people’s willingness to be tested [27, 28]. In light of these influences, we considered that relying on data from people who had been willing and able to access a test would limit both the generalizability of our findings and the potential sample size. Thus, we present here data relating to both whether people believed they had had the infection as well as whether they had been tested for infection.

In terms of symptoms, we asked respondents to report which of 11 symptoms (including the hallmark symptoms of persistent cough, fever, loss of taste, loss of smell) they had experienced and also to rate the severity of their overall symptom experience (from a scale of 1 to 10). From these we computed two symptom outcomes: total number of symptoms and a symptom severity score.

### Statistical Analysis

All analyses were performed using STATA (version 16). We used independent *t*-tests to compare differences in mean age and chi-squared tests to compare differences in gender, ethnicity, keyworker status, and COVID-19 risk status and psychological factors in the subcohorts included in the analyses and dropouts. To explore psychological predictors of COVID-19 infection and symptom experience, a series of multivariable linear and logistic regression analyses were performed. Our approach to measuring psychological responses to the pandemic allowed us to consider both state responses (i.e., those captured over a relatively short window) as well as more enduring psychological experience (i.e., experiences over longer periods of time). Thus, our approach to the analysis was to operationalize our psychological predictors in four ways in order to provide a comprehensive account of the data: (a) including psychological predictors at wave 1 only, (b) including psychological predictors at wave 2 only, (c) using aggregated (mean) psychological predictor scores from waves 1 and 2, and (d) using change scores from waves 1 to 2. All outcome variables were assessed during wave 3 and included: belief in having had COVID-19 (Y/N), having had a positive COVID-19 test results (Y/N), the number of symptoms experienced (0–11), and overall severity of symptoms (0–10). Depression and anxiety scores were square root transformed given evidence of substantial skew. Given the conceptual overlap of the multiple psychological factors measured (depression, anxiety, stress, perceived loneliness, positive mood, worry about getting COVID-19), we performed a principal components analysis to reduce the number of predictors entered into regression models based on wave 1 data. This indicated that three factors accounted for approximately 85% of the variance in these factors (see Supplementary Appendix S2). Examination of factor loadings indicated the three components extracted conceptually represented “distress” (higher depression, anxiety, stress, and lower positive mood), “worry about COVID-19” and “loneliness.” These factor loadings were then also applied to wave 2 data, to allow for averaging psychological experience over the two waves. Factor scores for these components were then added to regression models. These models controlled for age, gender, ethnicity (Black, Asian, and minority ethnic [BAME]/non-BAME [White British]), keyworker status (yes/no), and self-reported clinical risk group in line with UK government issued guidance during wave 1 (not at increased risk; at increased risk; at most increased risk). Regression models using individual psychological variables (as opposed to principal components) were also conducted and are presented in Supplementary Appendix S3. Further, we conducted additional analyses examining keyworker status as a potential moderator between psychological factors and COVID-19-related outcomes by repeating all regression models above but adding interaction terms (keyworker × variable) alongside main effects (Supplementary Appendix S4).

### Role of Sponsor

The study sponsor did not play a role in the study design, collection; analysis, and interpretation of data; in the writing of the report; or in the decision to submit the paper for publication.

## Results

### Cohort Characteristics

Table 1 summarizes the characteristics of the cohort analyzed in this paper, specifically those who contributed data during waves 1 and 3 and those who completed all three waves. The “waves 1 and 3 completers” subcohort had a mean age of 50 years (*SD* = 15), with 85% female (*n* = 928), 6% from the minority ethnic backgrounds. Forty-two percent (*n* = 458) were keyworkers and 24% (*n* = 257) identified themselves as having risk factors which would put them at increased or higher risk of COVID-19.

**Table 1.** Participant demographics^a^

	Completed W1 and W3	Completed all waves	Original cohort (completed W1)
	*n* (%)	*n* (%)	*n* (%)
*N*	1,087 (35.1%)	879 (28.4%)	3,097 (100%)
Gender			
Male	158 (14.5%)	123 (14.0%)	476 (15.4%)
Female	928 (85.4%)	755 (85.9%)	2,618 (84.5%)
Prefer not to say	1 (0.1%)	1 (0.1%)	3 (0.1%)
Age (mean, *SD*)	49.46 (15.0)	49.70 (15.0)	44.58 (15.0)
Age groups (years)			
18–24	72 (6.6%)	49 (5.6%)	364 (11.8%)
25–34	138 (12.7%)	118 (13.4%)	528 (17.1%)
35–44	181 (16.7%)	147 (16.7%)	637 (20.6%)
45–54	245 (22.5%)	193 (22.0%)	690 (22.3%)
55–64	272 (25.0%)	218 (24.8%)	570 (18.4%)
65–74	148 (13.6%)	129 (14.7%)	257 (8.3%)
≥75	31 (2.9%)	25 (2.8%)	49 (1.6%)
Ethnicity			
Non-BAME background	1,021 (93.9%)	827 (94.1%)	2,796 (90.3%)
BAME background	65 (6.0%)	51 (5.8%)	296 (9.6%)
Keyworker status			
Keyworker	458 (42.1%)	354 (40.3%)	1,559 (50.3%)
Nonkeyworker	629 (57.9%)	525 (59.7%)	1,538 (49.7%)
COVID-19 risk groups			
Most at risk (e.g., suffering from advanced cancer, severe asthma/COPD, etc.)	34 (3.1%)	25 (2.8%)	121 (3.9%)
At increased risk (e.g., being pregnant, aged over 70, etc.)	223 (20.5%)	180 (20.5%)	528 (17.1%)
Not at-risk	830 (76.4%)	674 (76.7%)	2,448 (79.0%)

*BAME* Black, Asian, and minority ethnic; COPD, Chronic obstructive pulmonary disease.

^a^W1: between 3/4/2020 and 30/4/2020, W2: between 1/7/20 and 21/9/20, W3: between 11/11/2020 and 31/12/2020.

The original cohort who participated in this research consisted of 3,097 participants at wave 1. Thus, for comparative purposes Table 1 also presents data on the relevant measures from the wider cohort at wave 1. *T*-Tests between the subcohort who completed both waves 1 and 3 compared with dropouts showed that those who completed waves 1 and 3 were significantly older(*t* = −13.7, *p* < .001); less likely to report a minority ethnic background (χ ^2^ = 24.9, *p* < .001), less likely to be a keyworker (χ ^2^ = 45.1, *p* < .001), and more likely to be in a COVID-19 risk group than the dropouts(χ ^2^ = 15.9, *p* < .001). Dropouts were also more likely to have higher levels of depression (*t* = 9.12, *p* < .001), anxiety (*t* = 8.97, *p* < .001), stress (*t* = 8.81, *p* < .001), loneliness (*t* = 6.37, *p* < .001), fear of getting COVID-19 (*t* = 2.45, *p* = .007) and lower positive mood (*t* = −6.68, *p* < .001). Similar nonrandom patterns of attrition were observed when comparing those who completed all three waves against those who did not.

### Psychological Predictors of COVID-19 Infection

Tables 2 and 3 present the mean scores for the psychological predictors and their intercorrelations, as well as the COVID-19 outcomes for those in the sample who completed wave 3. For composite scores from principal components analyses, there were significant correlations between waves 1 and 2 scores for the extracted components labeled “distress” (*r* = .72, *p* < .001), “worry” (*r* = .50, *p* < .001), and “loneliness” (*r* = .49, *p* < .001). For individual psychological variables, between waves 1 and 2 paired sample *t*-tests demonstrated decreases in depression (*t* = 4.93, *p* < .001), anxiety (*t* = 6.80, *p* < .001), stress (*t* = 3.95, *p* < .001), loneliness (*t* = 3.78, *p* < .001), and worry about COVID-19 levels (χ ^2^ = 524.5, *p* < .001), and increases in positive mood (*t* = −6.01, *p* < .001) coinciding with the easing of social restrictions in the UK during wave 2. The same pattern was observed when considering composite variable scores from principal components analyses with participants decreasing in distress (*t* = 7.73, *p* < .001), worry (*t* = 3.89, *p* < .001), and loneliness (*t* = 4.52, *p* < .001) from waves 1 to 2.

**Table 2.** Descriptions of psychological predictors and COVID-19 outcomes

	Completed W1 and W3 (*n* = 1,087)	Completed all waves (*n* = 879)	Original cohort (*n* = 3,097)
Psychological predictors at W1			
Depression—mean (*SD*), observed range	6.4 (5.5), 0–27	5.97 (5.19), 0–27	7.69 (6.0), 0–27
Anxiety—mean (*SD*), observed range	5.4 (5.1), 0–21	5.15 (4.96), 0–21	6.59 (5.6), 0–21
Stress—mean (*SD*), observed range	5.8 (3.2), 0–16	5.59 (3.10), 0–16	6.48 (3.3), 0–16
Positive mood—mean (*SD*), observed range	19.8 (5.0), 6–30	20.08 (4.89), 6–30	18.99 (5.1), 6–30
Loneliness—mean (*SD*), observed range	3.4 (2.6), 1–10	3.33 (2.55), 1–10	3.86 (2.7), 1–10
Worry about COVID-19 (*n*, %)			
No worry	182 (16.7%)	153 (17.4%)	512 (16.5%)
Occasionally worry	746 (68.6%)	605 (68.8%)	2,050 (66.2%)
Much of time	129 (11.9%)	102 (11.6%)	413 (13.3%)
Most of time	30 (2.8%)	19 (2.2%)	122 (3.9%)
Composite variables scores (PCA)			
“Distress”—mean (*SD*)	−0.44 (1.85)	−0.56 (1.79)	0.00 (1.91)
“Worry”	0.03 (0.88)	0.03 (0.87)	0.00 (0.94)
“Loneliness”	0.02 (0.74)	0.03 (0.73)	0.00 (0.76)
Psychological predictors at W2			
Depression—mean (*SD*), observed range	—	5.29 (4.90), 0–27	—
Anxiety—mean (*SD*), observed range	—	4.37 (4.55), 0–21	—
Stress—mean (*SD*), observed range	—	5.23 (3.14), 0–15	—
Positive mood—mean (*SD*), observed range	—	20.89 (4.96), 6–30	—
Loneliness—mean (*SD*), observed range	—	3.05 (2.44), 1–10	—
Worry about COVID-19 (*n*, %)	—		—
No worry	—	191 (21.7%)	
Occasionally worry	—	627 (71.3%)	—
Much of time	—	50 (5.7%)	—
Most of time	—	11 (1.3%)	—
Composite variables (PCA)			
“Distress”	—	−0.89 (1.80)	—
“Worry”	—	−0.08 (0.80)	—
“Loneliness”	—	0.02 (0.68)	—
COVID-19 outcomes at W3			
Belief of having had COVID-19 (*n*, %)	266 (24.5%)	204 (23.2%)	—
Having a positive test result (*n*, %)	34 (3.1%)	25 (2.8%)	—
Number of symptoms—mean (*SD*), observed range	1.9 (2.7), 0–11	1.55 (2.5), 0–10	—
Severity of symptoms—mean (*SD*), observed range	5.0 (2.0), 1–10	5.02 (2.01), 1–10	—

*PCA* principal components analysis. Depression (PHQ-9) scores have a possible range from 0 to 27. Anxiety (GAD-7) scores have a possible range from 0 to 21. Stress (PSS-4) scores have a possible range from 0 to 16. Positive mood (SPANE-P) scores have a possible range from 6 to 30. Loneliness (single item) and severity of symptoms (single item) have possible ranges of 1–10. Number of symptoms has a possible range of 0–11.

**Table 3.** Pearson’s correlations between psychological predictors

	(2)	(3)	(4)	(5)	(6)	(7)	(8)	(9)	(10)	(11)	(12)
(1) W1 Depression^a^	.78	.67	−.67	.55	.25	.65	.60	.52	−.48	.43	.17
(2) W1 Anxiety^a^		.68	−.64	.46	.37	.55	.68	.52	−.44	.37	.25
(3) W1 Stress			−.71	.52	.25	.46	.50	.61	−.49	.39	.20
(4) W1 Positive mood				−.53	−.29	−.47	−.48	−.55	.64	−.40	−.21
(5) W1 Loneliness					.17	.36	.33	.36	−.33	.60	.05
(6) W1 Worry about COVID-19						.13	.20	.17	−.17	.12	.50
(7) W2 Depression^a^							.77	.66	−.64	.54	.17
(8) W2 Anxiety^a^								.66	−.62	.49	.25
(9) W2 Stress									−.70	.53	.22
(10) W2 Positive mood										−.55	−.20
(11) W2 Loneliness											.16
(12) W2 Worry about COVID-19											—

Worry about COVID-19 was treated as a scale ranging from 1 to 4 with higher scores indicating greater levels of worry.

^a^Square-root transformed.

Analyses in which psychological variables measured during wave 1 only were included revealed that younger respondents (odds ratio [OR]: 0.86, 95% confidence interval [CI]: [0.77, 0.95]), keyworkers (OR: 1.37, 95% CI: [1.03, 1.82]), and those who at wave 1 reported greater distress (OR: 1.12, 95% CI: [1.03, 1.21]) were more likely at wave 3 to report believing they had had a COVID-19 infection (Table 4). Among participants who were tested for COVID-19 infection (*n* = 494), the direction of these relationships remained consistent, although none were independently statistically significant in predicting having a positive test result (Table 5). However, the statistical power of this analysis is relatively low given the small number of respondents who tested positive for COVID-19 (*n* = 34). In models including wave 2 psychological variables as predictors, the pattern of results was broadly the same as with wave 1 measures, with the exception that being a keyworker was no longer independently statistically significant, and that greater worry about COVID-19 at wave 2 was associated with a lower likelihood of respondents believing they had had a COVID-19 infection (OR: 0.62, 95% CI: [0.50, 0.76]).

**Table 4.** Multivariable logistic regression models examining psychological predictors of belief in having had COVID-19 infection

	Belief of having COVID-19			
	W1 scores	W2 scores	Aggregate W1 and W2 scores	Change from W1 to W2
	Odds ratio [95% CI]	Odds ratio [95% CI]	Odds ratio [95% CI]	Odds ratio [95% CI]
Age (per decade)	0.857**	0.868*	0.884*	0.805***
	[0.77, 0.95]	[0.77, 0.97]	[0.79, 0.99]	[0.72, 0.90]
Female	1.041	1.003	0.965	1.068
	[0.68, 1.58]	[0.62, 1.63]	[0.59, 1.56]	[0.66, 1.72]
BAME	0.740	0.736	0.677	0.812
	[0.40, 1.37]	[0.36, 1.50]	[0.33, 1.39]	[0.40, 1.64]
Keyworker	1.365*	1.176	1.227	1.207
	[1.03, 1.82]	[0.84, 1.64]	[0.88, 1.70]	[0.87, 1.67]
Risk group^a^				
At most increased risk	0.488	0.954	0.856	0.956
	[0.18, 1.30]	[0.34, 2.66]	[0.31, 2.40]	[0.35, 2.63]
At increased risk	0.932	1.027	1.04	1.018
	[0.62, 1.33]	[1.03, 1.24]	[0.67, 1.60]	[0.67, 1.55]
Distress^b^	1.115**	1.130*	1.151**	1.084
	[1.03, 1.21]	[1.03, 1.24]	[1.04, 1.27]	[0.96, 1.23]
Worry about COVID-19^b^	0.895	0.616***	0.631***	0.795*
	[0.76, 1.05]	[0.50, 0.76]	[0.50, 0.79]	[0.66, 0.96]
Loneliness^b^	0.872	0.850	0.816	0.989
	[0.72, 1.05]	[0.67, 1.07]	[0.63, 1.06]	[0.31, 1.06]
*N*	1,086	878	878	878
pseudo *R*^2^	0.032	0.055	0.048	0.026

*BAME* Black, Asian, and minority ethnic; *CI* confidence interval. Significant independent predictors in the model are highlighted in bold (*p* < .05).

^a^Reference group: “not at increased risk.”

^b^Factor scores from principal components analyses.

*p < .05.

**p < .01.

***p < .001.

**Table 5.** Multivariable logistic regression models examining psychological predictors of having had a positive COVID-19 test

	Having a positive test result^a^			
	W1 scores	W2 scores	Aggregate W1 and W2 scores	Change from W1 to W2
	Odds ratio [95% CI]	Odds ratio [95% CI]	Odds ratio [95% CI]	Odds ratio [95% CI]
Age (per decade)	0.787	0.839	0.833	0.753
	[0.59, 1.05]	[0.61, 1.16]	[0.60, 1.15]	[0.55, 1.04]
Female	1.031	1.116	1.020	1.281
	[0.34, 3.11]	[0.31, 4.03]	[0.28, 3.66]	[0.35, 4.68]
BAME	1.460	1.378	1.195	1.674
	[0.40, 5.32]	[0.29, 6.54]	[0.25, 5.65]	[0.34, 8.21]
Keyworker	1.379	1.110	1.173	1.073
	[0.66, 2.87]	[0.48, 2.58]	[0.51, 2.70]	[0.46, 2.50]
Risk group^b^				
At most increased risk	—	—	—	—
At increased risk	0.624	0.768	0.736	0.794
	[0.22, 1.96]	[0.22, 2.71]	[0.21, 2.59]	[0.22, 2.82]
Distress^c^	1.056	1.054	1.148	0.893
	[0.86, 1.30]	[0.83, 1.34]	[0.88, 1.49]	[0.67, 1.19]
Worry about COVID-19^c^	1.306	0.660	0.887	0.556*
	[0.89, 1.92]	[0.40, 1.10]	[0.52, 1.52]	**[0.35, 0.88]**
Loneliness^c^	0.760	0.879	0.880	1.186
	[0.48, 1.21]	[0.47, 1.63]	[0.45, 1.71]	[0.67, 2.11]
*N*	477	370	370	370
pseudo *R*^2^	0.034	0.034	0.025	0.052

*BAME* Black, Asian, and minority ethnic; *CI* confidence interval. Significant independent predictors in the model are highlighted in bold (*p* < .05).

^a^Restricted to those who self-reported being tested.

^b^Reference group: “not at increased risk.”

^c^Factor scores from principal components analyses.

*p < .05.

**p < .01.

***p < .001.

A similar pattern was observed when considering aggregate psychological experiences over waves 1 and 2. The only notable difference was that while younger respondents (OR: 0.88, 95% CI: [0.79, 0.99]) and those with greater aggregate distress (OR: 1.15, 95% CI: [1.04, 1.27]) were again more likely to report believing they had had a COVID-19 infection; being a keyworker was not a significantly independent predictor, and greater aggregate worry about COVID-19 was associated with lower likelihood of believing they had had a COVID-19 infection (OR: 0.63, 95% CI: [0.50, 0.79]). When considering change from waves 1 to 2, the only statistically significant psychological variable in the models for belief in having had COVID-19 and having a positive test result was worry about COVID-19, such that those whose worry increased were less likely to have believed they had COVID-19 (OR: 0.80, 95% CI: [0.66, 0.96]), or receive a positive COVID-19 test result (OR: 0.56, 95% CI: [0.35, 0.88]).

### Psychological Predictors of COVID-19 Symptoms

Among those who reported a belief in having had COVID-19 (at any time) when responding at wave 3, in models containing psychological predictors at wave 1 only, females (regression coefficient *B* = 0.91, 95% CI: [0.09, 1.73]), those who self-identified as being in a higher clinical risk group (*B* = 0.98, 95% CI: [0.25, 1.71]), and those with higher levels of distress at wave 1 were associated with a greater number of reported symptoms (*B* = 0.19, 95% CI: [0.03, 0.34] per unit increase in distress score) (Table 6). Considering symptom severity, older participants (per decade *B* = 0.20, 95% CI: [0.02, 0.39]), those in the clinically most at-risk group (*B* = 2.02, 95% CI: [0.24, 3.79]), and those with higher levels of distress at baseline (*B* = 0.22, 95% CI: [0.08, 0.36] per unit) reported more severe symptoms associated with their COVID-19 infection (Table 7). The same pattern of findings was seen in models containing psychological predictors at wave 2 only, with the exception that distress at wave 2 was not a significant independent predictor of number of symptoms and age was not a significant predictor of symptom severity.

**Table 6.** Multivariable regression models examining psychological predictors of number of symptoms in respondents who believed they had had COVID-19

	Number of symptoms			
	W1 scores	W2 scores	Aggregate W1 and W2 scores	Change from W1 to W2
	*B* [95% CI]	*B* [95% CI]	*B* [95% CI]	*B* [95% CI]
Age (per decade)	−0.012	−0.039	−0.046	−0.064
	[−0.22, 0.20]	[−0.27, 0.19]	[−0.28, 0.19]	[−0.29, 0.16]
Female	0.911*	0.972*	0.982*	1.029*
	[0.09, 1.73]	[0.04, 1.90]	[0.07, 1.90]	[0.09, 1.97]
BAME	−0.104	−0.008	−0.074	0.071
	[−1.30, 1.09]	[−1.39, 1.37]	[−1.44, 1.29]	[−1.35, 1.49]
Keyworker	0.056	0.241	0.210	0.233
	[−0.50, 0.61]	[−0.38, 0.86]	[−0.41, 0.82]	[−0.39, 0.86]
Risk group^a^				
Most at risk	0.049	−0.123	−0.103	0.164
	[−1.95, 2.05]	[−2.10, 1.86]	[−2.06, 1.86]	[−1.83, 2.16]
Increased risk	0.980**	1.243**	1.221**	1.428**
	[0.25, 1.71]	[0.42, 2.07]	[0.41, 2.04]	[0.61, 2.25]
Distress^b^	0.185*	0.181	0.236*	−0.092
	[0.03, 0.34]	[−0.001, 0.36]	[0.04, 0.43]	[−0.33, 0.14]
Worry about COVID-19^b^	0.222	0.317	0.387	−0.090
	[−0.05, 0.50]	[−0.07, 0.70]	[−0.01, 0.78]	[−0.44, 0.26]
Loneliness^b^	0.028	−0.097	−0.001	−0.180
	[−0.34, 0.40]	[−0.52, 0.33]	[−0.48, 0.48]	[−0.62, 0.26]
*N*	266	204	204	204
*R* ^2^	0.08	0.10	0.12	0.08

*BAME* Black, Asian, and minority ethnic; *CI* confidence interval.Significant independent predictors in the model are highlighted in bold (*p* < .05).

^a^Reference group: “not at increased risk.”

^b^Factor scores from principal components analyses.

*p < .05.

**p < .01.

***p < .001.

**Table 7.** Multivariable regression models examining psychological predictors of severity of COVID-19 symptoms in respondents who believed they had had COVID-19

	Severity of symptoms			
	W1 scores	W2 scores	Aggregate W1 and W2 scores	Change from W1 to W2
	*B* [95% CI]	*B* [95% CI]	*B* [95% CI]	*B* [95% CI]
Age (per decade)	0.203*	0.196	0.193	0.142
	[0.02, 0.39]	[−0.01, 0.40]	[−0.01, 0.40]	[−0.06, 0.34]
Female	0.055	0.016	−0.003	0.032
	[−0.67, 0.78]	[−0.80, 0.83]	[−0.82, 0.81]	[−0.81, 0.87]
BAME	0.052	−0.002	−0.101	0.062
	[−1.01, 1.12]	[−1.22, 1.21]	[−1.31, 1.11]	[−1.20, 1.33]
Keyworker	0.465	0.582	0.556*	0.573*
	[−0.02, 0.95]	[0.04, 1.13]	[0.01, 1.10]	[0.01, 1.13]
Risk group^a^				
Most at risk	2.017*	1.849*	1.901*	2.146*
	[0.24, 3.79]	[0.11, 3.59]	[0.16, 3.64]	[0.37, 3.92]
Increased risk	0.241	0.434	0.450	0.626
	[−0.41, 0.89]	[−0.29, 1.16]	[−0.27, 1.17]	[−0.10, 1.36]
Distress^b^	0.220**	0.248**	0.271**	0.043
	[0.08, 0.36]	[0.09, 0.41]	[0.10, 0.44]	[−0.17, 0.25]
Worry about COVID-19^b^	0.072	0.133	0.146	−0.067
	[−0.17, 0.32]	[−0.21, 0.47]	[−0.21, 0.50]	[−0.38, 0.24]
Loneliness^b^	0.145	0.140	0.115	0.079
	[−0.19, 0.48]	[−0.24, 0.52]	[−0.31, 0.54]	[−0.32, 0.47]
*N*	266	204	204	204
*R* ^2^	0.09	0.12	0.12	0.07

*BAME* Black, Asian, and minority ethnic; *CI* confidence interval.

^a^Reference group: “not at increased risk.”

^b^Factor scores from principal components analyses.

*p < .05.

**p < .01.

***p < .001.

Analyses examining aggregate psychological experiences over waves 1 and 2 showed the same pattern of results as observed for models with wave 1 predictors only. Change in psychological variables from waves 1 to 2 did not predict symptom severity or number of symptoms in those who reported having had COVID-19 (at any time) when responding at wave 3.

**Fig. 1.**
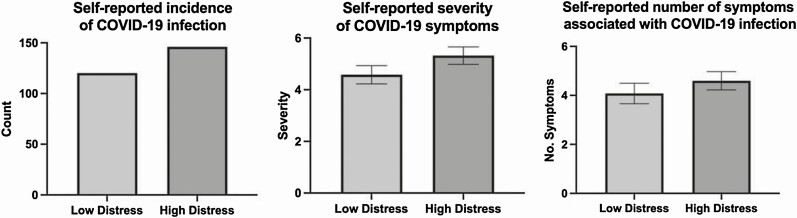
Self-reported COVID-19 outcomes based on median split of distress at baseline.

Figure 1 further illustrates the relationship between distress on COVID-19 infection and symptom experience by comparing high and low distress groups created using a median split of distress scores at baseline.

### Interactions With Keyworker Status

We conducted additional analyses examining keyworker status as a potential moderator between psychological factors and COVID-19-related outcomes for all models (see Supplementary Appendix for model results). In both PCA derived composite measures and original psychological models, no interaction terms were statistically significant, indicating the above relationships did not substantially differ between keyworkers and non-keyworkers.

## Discussion

We report here findings from a prospective cohort study established early in the course of the COVID-19 pandemic in the UK. Our aim was to examine whether, as has been demonstrated in the context of other viral infections, psychological factors were related to an individual’s risk of developing COVID-19 and the severity of their symptom experience. To our knowledge, this is the first study to demonstrate a small but significant effect of psychological distress on both the likelihood of reporting COVID-19 infection and the symptom experience. Specifically, we observed that, after controlling for sociodemographic factors known to be associated with an increased risk of infection, individuals who reported greater levels of distress during April 2020, during July–September 2020, and aggregated measures across those time points, were more likely to subsequently report having had COVID-19 and a greater number of symptoms which they also reported as being more severe.

These findings raise several issues worthy of further discussion. First, they suggest, as indicated by the original seminal viral challenge studies [12], that some of the variability in who develops COVID-19 and the severity of the condition, may be related to psychological distress, which in this work has been operationalized as a constellation of increased stress, anxiety, and depression and low levels of positive mood. This in turn raises questions about whether comparable effects may be observed for COVID-19 vaccines that is, might the effectiveness of these vaccines in protecting against disease also be influenced by psychological well-being? [29] As the success of COVID-19 vaccines will play a critical role in the route out of this pandemic, understanding the role of psychological factors in vaccine effectiveness, and the potential for psychological interventions to enhance effectiveness, could be important areas for future research [30, 31].

Second, the findings raise questions about mechanisms. Do these data suggest that acute distress associated with the early stages of the pandemic may influence susceptibility to COVID-19? Or that individual differences in distress more generally are related to vulnerability to infection? Though these data do not allow us to directly answer this question, we suggest that the latter is the more likely explanation. While our psychological measures at each wave were phrased in such a way as to capture relatively short-term experiences, and thus were amenable to change over time, we observed considerable intraindividual stability in our measures over the three data collection waves. Our distress component, for example, correlated highly across waves 1 and 2 meaning our wave 1 measure of distress likely captured both something about acute/state experience and more enduring individual differences. Our analyses looking at wave 2 data only, and aggregating psychological experiences over waves 1 and 2 data collection (in April 2020 and July–September 2020, respectively), also showed a very similar pattern of results to those just using wave 1 data alone. Together, these findings suggest the relationships reported here are more likely to be related to longer-term distress.

As to the mechanisms underlying this relationship between distress and COVID-19 outcomes, while further supporting evidence of this relationship is needed, previous research indicates there are plausible pathways for this relationship that could be behavioral or involve the sympathetic and hypothalamic–adrenal pathways [25]. In terms of behavior, health behaviors (e.g., smoking, alcohol consumption, sleep) have been shown to influence vulnerability to viral infections [25]. Indeed, several such behaviors have been found to be adversely affected during the COVID-19 pandemic with evidence, for example, of increased alcohol intake, poorer sleep quality, etc. [32–35]. Thus, it is plausible that changes in behaviors, related to or independent from psychological well-being, may increase the risk of COVID-19 infection.

There are also biologically plausible mechanisms that could underlie this relationship. The role of the sympathetic and hypothalamic–adrenal pathways in dysregulating the immune system and in turn influencing vulnerability to viral infections is well documented [8]. But of particular relevance is recent evidence which suggests a particular role for the hypothalamic–pituitary–adrenal axis. For example, a study of 535 patients admitted to hospital revealed that patients who went on to have a diagnosis of COVID-19 were also found to have higher levels of serum cortisol on admission. Furthermore, higher cortisol concentrations were also found to be associated with increased mortality in this cohort [36]. Of course, we cannot determine from such evidence whether the increased levels of cortisol seen in people with COVID-19 were due to increased psychological distress, potentially increasing the risk of infection, or indeed part of the pathogenic process underlying the disease. But the data signal that elevated levels of the hormone are associated with greater risk of having COVID-19 and a poorer outcome and permit us to hypothesize that the release of cortisol in response to psychological distress could both increase the risk of COVID-19 infection, through immune suppression, but also lead to poorer outcomes through ongoing dysregulation of the immune response to the disease.

In addition to the effects of distress, we observed that individuals who worried more about getting COVID-19 at wave 2, had greater aggregated levels of worry over both waves, and increased more in worry from waves 1 to 2 were less likely to report having had COVID-19. This could be attributable to a behavioral pathway in which those most worried about getting COVID-19 engaged in greater preventative behaviors and thus were less likely to become infected. However, our supplementary analyses examining individual psychological variables (as opposed to composite variables), indicated that compared with those who only “occasionally worried” about COVID-19, those who worried “all of the time” about COVID-19 were also more likely to have had a positive test result by December 2020 (as well as those who “did not worry”). This suggests that both high and low levels of COVID-19-related worry may be associated with greater risk of COVID-19 infection, although further research is needed to disentangle these effects.

A third observation concerns the absence of a significant effect of perceived loneliness on risk of developing COVID-19 or the experience of symptoms. This initially appears surprising given the wealth of evidence suggesting a role for loneliness and other indices of social support in health outcomes including in viral infections [8, 25]. One explanation for these findings may be related to the time at which loneliness was assessed in this study that is, within the first few weeks of the UKs first national lockdown. It is conceivable that experiences of loneliness may not have manifested themselves at this early stage, as evidenced by the low levels of loneliness in the cohort as a whole (see Table 2) thus reducing their potential impact. Analyses including wave 2 may also have failed to capture the height of loneliness within the cohort, as during this time restrictions had eased somewhat—allowing greater social interactions. While conversely, high levels of psychological distress (such as high levels of depression and anxiety) were evident in the cohort, even at this early stage of the pandemic, with observed levels significantly exceeding those previously reported for a general public cohort in the UK [2]. We also note that our measures of loneliness (and also worry about getting COVID-19) were based on single item measures, rather than validated scales, which may reduce the reliability of these measures and thus the confidence that can be placed in findings relating to those variables.

Although this study includes a number of strengths including the large sample size, the prospective design and inclusion of individuals largely representative of the UK population. A number of possible limitations should also be acknowledged. First, all our COVID-19 outcomes were self-reported. There is, therefore, an urgent need to examine whether our results are replicated in cohorts where data on verified cases of infection are available. The reliance on self-reported outcomes also brings the potential issue of reverse causality, with some respondents who may have experienced infection early in the pandemic potentially being more likely to report higher distress later on. We tried to minimize this possibility and response biases in our analysis approach by focusing our primary analyses on psychological predictors measured during April 2020 and using outcomes collected in November–December 2020. However, given first peak of infection is estimated to have occurred during the period participants were recruited into the cohort (April 2020), it is probable that some infection instances were prior to wave 1 data collection, and indeed many will have likely occurred prior to wave 2 data collection. A second, and related issue is that in terms of our COVID-19 infection outcome, our main analysis relied on people’s belief that they had had the infection. The reasons for doing so have been articulated above. But further justification for our approach includes the fact that the classic symptoms of COVID-19 are universally recognized. Thus, participants were potentially able to determine with some confidence if they had had COVID-19. In addition, the analysis focusing only on people who reported having a COVID-19 test, produced comparable results (albeit findings were not statistically significant most likely due to the modest size of this subgroup). Notwithstanding these considerations, the absence of verified infection necessarily means we cannot account for the large numbers of individuals for whom the infection is asymptomatic [23], even though they would of course be less likely to seek a test. Thus, future work focusing on laboratory assessment of antibodies to SARS-CoV-2 and/or verified test results would permit greater confidence in the associations described here.

Third, we observed significant, nonrandom, attrition from the cohort. Although the degree of attrition was comparable to that reported in other cohorts established early in the pandemic [37, 38], we saw greater dropout among those who reported poorer psychological well-being at baseline and keyworkers, who were arguably the most risk exposed to COVID-19 infection. This may mean that the relationships observed between psychological distress and COVID-19 infection outcomes underestimate the true effects in the population, given infection outcomes for those with the greatest distress were less likely to be represented in the models. It is therefore important that these findings are interpreted with appropriate caution, given the potential for such attrition to result in a systematic selection bias [39].

In summary, we report here evidence from a large prospective cohort study which demonstrates that, even after controlling for known demographic risk factors for COVID-19 infection and the imprecision of our measure of COVID-19 infection, psychological distress was associated with a small but significant increase in the risk of COVID-19 infection and an increase in the number and severity of symptoms.

## Supplementary Material

kaab106_suppl_Supplementary_Material
