# Supplementary material for: Psychological Predictors of Self-reported COVID-19 Outcomes: Results From a Prospective Cohort Study
Source: Ann Behav Med. 2022 Jan 3;56(5):484–97. doi: 10.1093/abm/kaab106 (PMC8755370; doi:10.1093/abm/kaab106)
Supplement: kaab106_suppl_Supplementary_Material [file kaab106_suppl_supplementary_material.docx]

**Supplementary Appendix**

**Appendix S1: Details of measures**

|  | **Question/scale** | **Response(s)** |
| --- | --- | --- |
| **Demographic factors** |  |  |
| Gender* | What was your gender at birth? | Male |
|  |  | Female |
|  |  | Other |
|  |  | Prefer not to say |
| Age | How old are you? | .. |
| Ethnicity* | What is your ethnicity | White – British, Irish, other |
|  |  | Asian/Asian British – Indian, Pakistani, Bangladeshi, other |
|  |  | Black/Black British – Caribbean, African, other |
|  |  | Chinese/Chinese British |
|  |  | Mixed race – White and Black/Black British |
|  |  | Middle Eastern/Middle Eastern British – Arab, Turkish, other |
|  |  | Mixed race – other |
|  |  | Other ethnic group |
|  |  | Prefer not to say |
| Key-worker status | Are you currently fulfilling any of the government’s identified ‘key worker’ roles? | Health, social care or relevant related support worker |
|  |  | Teacher or childcare worker still travelling in to work |
|  |  | Transport worker still travelling in to work |
|  |  | Food chain worker (e.g. production, sale, delivery) |
|  |  | Key public services worker (e.g. justice staff, religious staff, public service journalist or mortuary worker) |
|  |  | Local or national government worker delivering essential public services |
|  |  | Utility worker (e.g. energy, sewerage, postal service) |
|  |  | Public safety or national security worker |
|  |  | Worker involved in medicines or protective equipment production or distribution |
|  |  | Other ‘key worker’ role not listed |
|  |  | None of these |
| COVID-19 risk status | Which of these 3 COVID-19 risk groups do you think you are in? | I am most at risk (e.g. suffering from advanced cancer, severe asthma/COPD, etc.) |
|  |  | I am at increased risk (e.g. being pregnant, aged over 70, etc.) |
|  |  | I am in neither risk category |
| **Psychological factors** |  |  |
| *Perceived loneliness^†^* | On a scale of 1-10, how lonely have you felt over the past 2 weeks? | 1 (Not at all lonely) - 10 (Extremely lonely) |
| *Worry about contracting COVID-19* | Please read the following statements carefully and then select the one which best describe how you have felt over the past 2 weeks. | I do not worry about getting COVID-19. |
|  |  | I occasionally worry about getting COVID-19. |
|  |  | I spend much of my time worrying about getting COVID-19. |
|  |  | I spend most of my time worrying about getting COVID-19. |
| **COVID-19 outcomes** |  |  |
| COVID-19 Test | Since the beginning of the pandemic, have you had a COVID-19 test at any point? By this we mean the test for whether you have COVID-19 at the time, not an antibody test. | Yes |
|  |  | No |
| Tested positive for COVID-19 | (If answered yes to the COVID-19 test) Thinking about the test result(s), have you tested positive for COVID-19 at any point? | Yes |
|  |  | No |
| COVID-19 symptoms | (If answered yes to the COVID-19 positive test) Thinking about your most recent positive test, did you have any of these COVID-19 related symptoms? (Please select all that apply) | Persistent cough |
|  |  | Fatigue |
|  |  | Shortness of breath |
|  |  | Loss of smell |
|  |  | Loss of taste |
|  |  | Sore throat |
|  |  | Tightness in chest |
|  |  | Diarrhoea |
|  |  | Loss of appetite |
|  |  | Feeling confused |
|  |  | Fever |
|  |  | None of the above |
| COVID-19 symptom severity | (If answered yes to COVID-19 positive test) Thinking about your most recent positive test, how severe were your symptoms ( on a scale of 1-10)? | 1 (I didn’t have any symptoms) – 10 (very severe) |
| Belief of having COVID-19 | Regardless of your previous test(s), do you believe that you have had COVID-19 previously? | Yes |
|  |  | No |
| Belief of COVID-19 symptoms | (If answered yes to belief of having COVID-19) Thinking about your most recent positive test, did you have any of these COVID-19 related symptoms? (Please select all that apply) | Persistent cough |
|  |  | Fatigue |
|  |  | Shortness of breath |
|  |  | Loss of smell |
|  |  | Loss of taste |
|  |  | Sore throat |
|  |  | Tightness in chest |
|  |  | Diarrhoea |
|  |  | Loss of appetite |
|  |  | Feeling confused |
|  |  | Fever |
|  |  | None of the above |
| Belief of COVID-19 symptom severity | (If answered yes to belief of having COVID-19) Thinking about your most recent positive test, how severe were your symptoms ( on a scale of 1-10)? | 1 (I didn’t have any symptoms) – 10 (very severe) |

*Gender and ethnicity were treated as binary variables in all analyses: gender (male, female), ethnicity (white British, non-white British).

^†^ The factors in *Italic* were hypothesised to be associated with an increased risk of adverse mental health outcomes, apart from key-worker status where evidence exists that some key-worker roles are also associated with an increased risk of adverse COVID-19 outcomes. All other factors were hypothesised to be associated with an increased risk of contracting COVID-19 and/or poorer disease outcomes.

^‡^Positive mood was measured using the positive items from SPANE: Scale of Positive and Negative Experience (α=0.94).

**Appendix S2 – PCA loadings**

**Supplementary Table : PCA Eigenvalues & Proportions**

|  | **Eigen Value** | **Proportion** | **Cumulative** |
| --- | --- | --- | --- |
| **Component 1** | 3.63578 | 0.6060 | 0.6060 |
| **Component 2** | .892977 | 0.1488 | 0.7548 |
| **Component 3** | .572457 | 0.0954 | 0.8502 |
| **Component 4** | .414721 | 0.0691 | 0.9193 |
| **Component 5** | .282184 | 0.0470 | 0.9664 |
| **Component 6** | 201883 | 0.0336 | 1.0000 |

**Supplementary Table: PCA Factor Loadings**

| **Variable** | **Factor 1 (Distress)** | **Factor 2 (Worry)** | **Factor 3 (Loneliness)** | **Factor 4** | **Factor 5** | **Factor 6** |
| --- | --- | --- | --- | --- | --- | --- |
| **Depression** | **0.4587** | -0.1186 | -0.1924 | **0.4712** | -0.2455 | **0.6754** |
| **Anxiety** | **0.4536** | 0.0859 | -0.3367 | **0.4548** | 0.0746 | **-0.6791** |
| **Stress** | **0.4490** | -0.1243 | -0.1952 | **-0.4228** | **0.7310** | 0.1781 |
| **Loneliness** | 0.3667 | -0.2802 | **0.8657** | 0.1352 | 0.0662 | -0.1219 |
| **Positive Mood** | **-0.4450** | 0.0782 | 0.0910 | **0.6098** | **0.6283** | 0.1449 |
| **Worry about COVID-19** | 0.2229 | **0.9373** | 0.2318 | -0.0486 | 0.0264 | 0.1228 |

Loadings with an absolute value >.4 are highlighted

**Supplementary Figure : PCA Scree Plot**

**
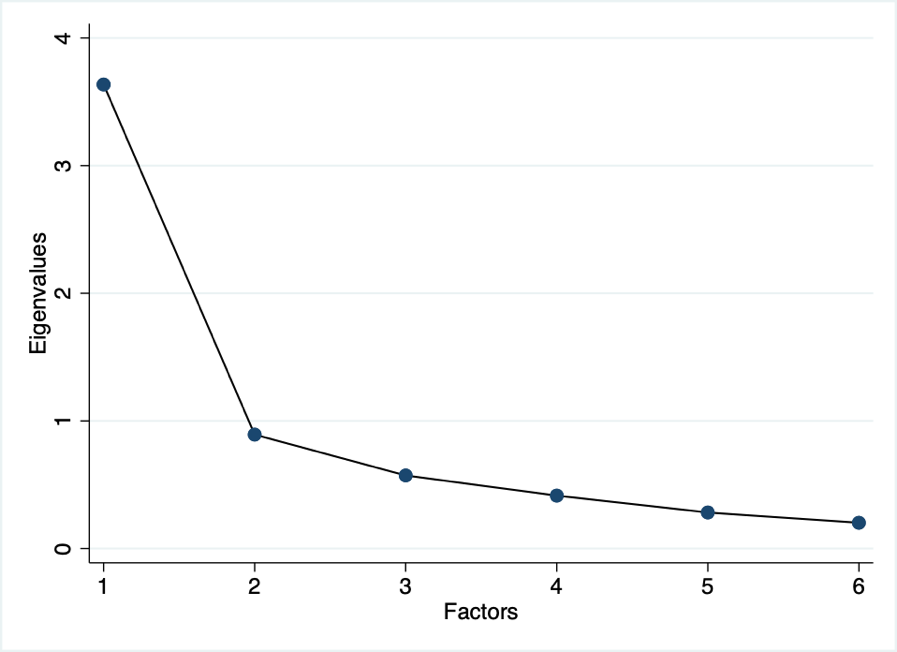
**

**Appendix S3 – Individual Psychological Variable Regression Models**

**Wave 1 Scores**

Supplementary Table: Models predicting belief of having had COVID-19 when asked at wave 3 (Odds Ratios)

|  | (1) | (2) | (3) | (4) | (5) | (6) | (7) |
| --- | --- | --- | --- | --- | --- | --- | --- |
| **Age (per decade)** | 0.870^**^ | 0.848^**^ | 0.826^***^ | 0.825^***^ | 0.828^***^ | 0.827^***^ | 0.883^*^ |
| **Female** | 1.019 | 1.042 | 1.078 | 1.072 | 1.076 | 1.088 | 1.017 |
| **BAME** | 0.771 | 0.764 | 0.754 | 0.739 | 0.756 | 0.760 | 0.786 |
| **Keyworker** | 1.312 | 1.337^*^ | 1.375^*^ | 1.368^*^ | 1.367^*^ | 1.369^*^ | 1.299 |
| **Risk Group^⊥^** |  |  |  |  |  |  |  |
| *At most increased risk* | 0.442 | 0.487 | 0.504 | 0.494 | 0.530 | 0.502 | 0.477 |
| *At increased risk* | 0.887 | 0.908 | 0.927 | 0.929 | 0.933 | 0.914 | 0.907 |
| **Depression** | 1.292^***^ |  |  |  |  |  | 1.360^**^ |
| **Anxiety** |  | 1.185^**^ |  |  |  |  | 1.041 |
| **Stress** |  |  | 1.032 |  |  |  | 0.955 |
| **Positive Mood** |  |  |  | 0.968^*^ |  |  | 0.989 |
| **Worry about COVID-19** |  |  |  |  |  |  |  |
| No worry |  |  |  |  | 1.565^*^ |  | 1.618^*^ |
| Some of time |  |  |  |  | 1.303 |  | 1.064 |
| Most of time |  |  |  |  | 1.773 |  | 1.217 |
| **Loneliness** |  |  |  |  |  | 1.035 | 0.979 |
| *N* | 1086 | 1086 | 1086 | 1086 | 1086 | 1086 | 1086 |
| pseudo *R*^2^ | 0.036 | 0.029 | 0.024 | 0.027 | 0.029 | 0.024 | 0.043 |

Supplementary Table: Models predicting positive COVID-19 test result when asked at wave 3 (Odds Ratios)

|  | (1) | (2) | (3) | (4) | (5) | (6) | (7) |
| --- | --- | --- | --- | --- | --- | --- | --- |
| **Age (per decade)** | 0.819 | 0.828 | 0.822 | 0.810 | 0.802 | 0.790 | 0.771 |
| **Female** | 1.064 | 1.055 | 1.060 | 1.071 | 1.101 | 1.107 | 1.109 |
| **BAME** | 1.337 | 1.319 | 1.271 | 1.257 | 1.371 | 1.426 | 1.333 |
| **Keyworker** | 1.453 | 1.446 | 1.463 | 1.469 | 1.447 | 1.441 | 1.416 |
| **Risk Group^⊥^** |  |  |  |  |  |  |  |
| *At most increased risk* | - | - | - | - | - | - | - |
| *At increased risk* | 0.665 | 0.659 | 0.671 | 0.673 | 0.631 | 0.677 | 0.651 |
| **Depression** | 1.064 |  |  |  |  |  | 0.880 |
| **Anxiety** |  | 1.128 |  |  |  |  | 1.076 |
| **Stress** |  |  | 1.046 |  |  |  | 1.058 |
| **Positive Mood** |  |  |  | 0.975 |  |  | 0.984 |
| **Worry about COVID-19** |  |  |  |  |  |  |  |
| No worry |  |  |  |  | 1.397 |  | 1.469 |
| Some of time |  |  |  |  | 1.693 |  | 1.701 |
| Most of time |  |  |  |  | 4.634^*^ |  | 4.911^*^ |
| **Loneliness** |  |  |  |  |  | 0.942 | 0.885 |
| *N* | 477 | 477 | 477 | 477 | 477 | 477 | 477 |
| pseudo *R*^2^ | 0.020 | 0.022 | 0.022 | 0.021 | 0.038 | 0.022 | 0.049 |

Supplementary Table: Models predicting number of COVID-19 symptoms when asked at wave 3 (standardised Betas)

|  | (1) | (2) | (3) | (4) | (5) | (6) | (7) |
| --- | --- | --- | --- | --- | --- | --- | --- |
| **Age (per decade)** | 0.042 | 0.021 | 0.000 | -0.007 | -0.039 | 0.005 | 0.004 |
| **Female** | 0.122^*^ | 0.136^*^ | 0.141^*^ | 0.143^*^ | 0.132^*^ | 0.141^*^ | 0.105 |
| **BAME** | -0.005 | -0.006 | -0.004 | -0.006 | 0.001 | 0.002 | 0.015 |
| **Keyworker** | 0.015 | 0.024 | 0.031 | 0.028 | 0.014 | 0.034 | -0.000 |
| **Risk Group^⊥^** |  |  |  |  |  |  |  |
| *At most increased risk* | 0.003 | 0.007 | 0.003 | 0.006 | 0.006 | 0.003 | -0.003 |
| *At increased risk* | 0.165^**^ | 0.167^**^ | 0.179^**^ | 0.182^**^ | 0.174^**^ | 0.176^**^ | 0.151^*^ |
| **Depression** | 0.232^***^ |  |  |  |  |  | 0.396^***^ |
| **Anxiety** |  | 0.115 |  |  |  |  | -0.127 |
| **Stress** |  |  | 0.062 |  |  |  | -0.061 |
| **Positive Mood** |  |  |  | -0.063 |  |  | 0.133 |
| **Worry about COVID-19** |  |  |  |  |  |  |  |
| No worry |  |  |  |  | -0.079 |  | -0.122 |
| Some of time |  |  |  |  | 0.022 |  | -0.005 |
| Most of time |  |  |  |  | 0.144^*^ |  | 0.104 |
| **Loneliness** |  |  |  |  |  | 0.079 | 0.047 |
| *N* | 266 | 266 | 266 | 266 | 266 | 266 | 266 |
| *R*^2^ | 0.101 | 0.063 | 0.055 | 0.055 | 0.080 | 0.057 | 0.142 |
| Adj. *R*^2^ | 0.077 | 0.038 | 0.029 | 0.029 | 0.047 | 0.032 | 0.094 |

Supplementary Table: Models predicting COVID-19 symptom severity when asked at wave 3 (standardised Betas)

|  | (1) | (2) | (3) | (4) | (5) | (6) | (7) |
| --- | --- | --- | --- | --- | --- | --- | --- |
| **Age (per decade)** | 0.151^*^ | 0.144^*^ | 0.138^*^ | 0.123^*^ | 0.090 | 0.141^*^ | 0.118 |
| **Female** | 0.004 | 0.013 | 0.015 | 0.019 | 0.011 | 0.016 | 0.004 |
| **BAME** | 0.010 | 0.008 | 0.004 | -0.003 | 0.017 | 0.018 | 0.013 |
| **Keyworker** | 0.110 | 0.115 | 0.125^*^ | 0.118 | 0.109 | 0.129^*^ | 0.114 |
| **Risk Group^⊥^** |  |  |  |  |  |  |  |
| *At most increased risk* | 0.139^*^ | 0.142^*^ | 0.132^*^ | 0.138^*^ | 0.147^*^ | 0.135^*^ | 0.135^*^ |
| *At increased risk* | 0.048 | 0.046 | 0.058 | 0.068 | 0.060 | 0.053 | 0.063 |
| **Depression** | 0.178^**^ |  |  |  |  |  | 0.127 |
| **Anxiety** |  | 0.119 |  |  |  |  | -0.110 |
| **Stress** |  |  | 0.153^*^ |  |  |  | 0.032 |
| **Positive Mood** |  |  |  | -0.179^**^ |  |  | -0.064 |
| **Worry about COVID-19** |  |  |  |  |  |  |  |
| No worry |  |  |  |  | -0.017 |  | -0.029 |
| Some of time |  |  |  |  | 0.037 |  | 0.019 |
| Most of time |  |  |  |  | 0.159^*^ |  | 0.111 |
| **Loneliness** |  |  |  |  |  | 0.153^*^ | 0.079 |
| *N* | 266 | 266 | 266 | 266 | 266 | 266 | 266 |
| *R*^2^ | 0.080 | 0.064 | 0.073 | 0.082 | 0.077 | 0.073 | 0.106 |
| Adj. *R*^2^ | 0.055 | 0.038 | 0.048 | 0.057 | 0.044 | 0.048 | 0.056 |

**Wave 2 Scores**

Supplementary Table: Models predicting belief of having had COVID-19 when asked at wave 3 (Odds Ratios)

|  | (1) | (2) | (3) | (4) | (5) | (6) | (7) |
| --- | --- | --- | --- | --- | --- | --- | --- |
| **Age (per decade)** | 0.867^*^ | 0.844^**^ | 0.840^**^ | 0.825^***^ | 0.818^***^ | 0.835^**^ | 0.870^*^ |
| **Female** | 0.942 | 0.981 | 0.978 | 1.021 | 1.094 | 1.003 | 1.001 |
| **BAME** | 0.783 | 0.754 | 0.734 | 0.739 | 0.745 | 0.718 | 0.762 |
| **Keyworker** | 1.233 | 1.235 | 1.244 | 1.250 | 1.205 | 1.271 | 1.180 |
| **Risk Group^⊥^** |  |  |  |  |  |  |  |
| *At most increased risk* | 0.719 | 0.834 | 0.791 | 0.768 | 1.094 | 0.775 | 0.903 |
| *At increased risk* | 0.952 | 0.998 | 0.977 | 0.977 | 1.095 | 0.966 | 1.016 |
| **Depression** | 1.361^***^ |  |  |  |  |  | 1.355^*^ |
| **Anxiety** |  | 1.196^*^ |  |  |  |  | 0.903 |
| **Stress** |  |  | 1.097^***^ |  |  |  | 1.055 |
| **Positive Mood** |  |  |  | 0.948^**^ |  |  | 0.986 |
| **Worry about COVID-19** |  |  |  |  |  |  |  |
| No worry |  |  |  |  | 1.700^**^ |  | 1.853^**^ |
| Some of time |  |  |  |  | 0.654 |  | 0.497 |
| Most of time |  |  |  |  | 0.364 |  | 0.199 |
| **Loneliness** |  |  |  |  |  | 1.089^**^ | 1.005 |
| *N* | 878 | 878 | 878 | 878 | 878 | 878 | 878 |
| pseudo *R*^2^ | 0.036 | 0.025 | 0.031 | 0.029 | 0.030 | 0.025 | 0.058 |

Supplementary Table: Models predicting positive COVID-19 test result when asked at wave 3 (Odds Ratios)

|  | (1) | (2) | (3) | (4) | (5) | (6) | (7) |
| --- | --- | --- | --- | --- | --- | --- | --- |
| **Age (per decade)** | 0.782 | 0.821 | 0.799 | 0.794 | 0.795 | 0.802 | 0.753 |
| **Female** | 1.123 | 1.047 | 1.084 | 1.095 | 1.193 | 1.124 | 1.188 |
| **BAME** | 1.249 | 1.329 | 1.258 | 1.350 | 1.256 | 1.325 | 1.318 |
| **Keyworker** | 1.175 | 1.168 | 1.176 | 1.236 | 1.181 | 1.201 | 1.310 |
| **Risk Group^⊥^** |  |  |  |  |  |  |  |
| *At most increased risk* | - | - | - | - | - | - | - |
| *At increased risk* | 0.767 | 0.726 | 0.739 | 0.697 | 0.875 | 0.728 | 0.926 |
| **Depression** | 0.956 |  |  |  |  |  | 0.519 |
| **Anxiety** |  | 1.207 |  |  |  |  | 1.629 |
| **Stress** |  |  | 1.039 |  |  |  | 0.945 |
| **Positive Mood** |  |  |  | 0.926 |  |  | 0.856^*^ |
| **Worry about COVID-19** |  |  |  |  |  |  |  |
| No worry |  |  |  |  | 1.367 |  | 1.742 |
| Some of time |  |  |  |  | - |  | - |
| Most of time |  |  |  |  | - |  | - |
| **Loneliness** |  |  |  |  |  | 1.066 | 0.984 |
| *N* | 370 | 370 | 370 | 370 | 345 | 370 | 345 |
| pseudo *R*^2^ | 0.017 | 0.022 | 0.018 | 0.034 | 0.020 | 0.020 | 0.075 |

Supplementary Table: Models predicting number of COVID-19 symptoms when asked at wave 3 (standardised Betas)

|  | (1) | (2) | (3) | (4) | (5) | (6) | (7) |
| --- | --- | --- | --- | --- | --- | --- | --- |
| **Age (per decade)** | 0.008 | -0.006 | -0.016 | -0.031 | -0.047 | -0.027 | -0.016 |
| **Female** | 0.151^*^ | 0.152^*^ | 0.155^*^ | 0.154^*^ | 0.139^*^ | 0.152^*^ | 0.130 |
| **BAME** | 0.023 | 0.008 | 0.011 | 0.007 | -0.005 | 0.010 | 0.025 |
| **Keyworker** | 0.044 | 0.049 | 0.054 | 0.059 | 0.074 | 0.060 | 0.061 |
| **Risk Group^⊥^** |  |  |  |  |  |  |  |
| *At most increased risk* | -0.008 | 0.003 | 0.002 | 0.005 | -0.007 | 0.005 | -0.022 |
| *At increased risk* | 0.218^**^ | 0.226^**^ | 0.230^**^ | 0.241^***^ | 0.223^**^ | 0.236^**^ | 0.204^**^ |
| **Depression** | 0.180^*^ |  |  |  |  |  | 0.326^**^ |
| **Anxiety** |  | 0.097 |  |  |  |  | -0.118 |
| **Stress** |  |  | 0.091 |  |  |  | 0.021 |
| **Positive Mood** |  |  |  | -0.040 |  |  | 0.118 |
| **Worry about COVID-19** |  |  |  |  |  |  |  |
| No worry |  |  |  |  | -0.140^*^ |  | -0.151^*^ |
| Some of time |  |  |  |  | 0.051 |  | 0.043 |
| Most of time |  |  |  |  | -0.028 |  | -0.061 |
| **Loneliness** |  |  |  |  |  | 0.034 | -0.028 |
| *N* | 204 | 204 | 204 | 204 | 204 | 204 | 204 |
| *R*^2^ | 0.107 | 0.085 | 0.085 | 0.079 | 0.100 | 0.078 | 0.143 |
| Adj. *R*^2^ | 0.075 | 0.053 | 0.052 | 0.046 | 0.059 | 0.045 | 0.079 |

Supplementary Table: Models predicting COVID-19 symptom severity when asked at wave 3 (standardised Betas)

|  | (1) | (2) | (3) | (4) | (5) | (6) | (7) |
| --- | --- | --- | --- | --- | --- | --- | --- |
| **Age (per decade)** | 0.147^*^ | 0.137 | 0.140 | 0.113 | 0.094 | 0.135 | 0.141 |
| **Female** | 0.001 | 0.002 | 0.010 | 0.012 | -0.011 | 0.008 | -0.007 |
| **BAME** | 0.018 | 0.002 | 0.007 | -0.004 | -0.008 | 0.008 | 0.019 |
| **Keyworker** | 0.129 | 0.133 | 0.135^*^ | 0.144^*^ | 0.167^*^ | 0.152^*^ | 0.165^*^ |
| **Risk Group^⊥^** |  |  |  |  |  |  |  |
| *At most increased risk* | 0.149^*^ | 0.159^*^ | 0.153^*^ | 0.155^*^ | 0.152^*^ | 0.155^*^ | 0.133 |
| *At increased risk* | 0.097 | 0.103 | 0.099 | 0.123 | 0.113 | 0.101 | 0.087 |
| **Depression** | 0.198^**^ |  |  |  |  |  | 0.224 |
| **Anxiety** |  | 0.128 |  |  |  |  | -0.148 |
| **Stress** |  |  | 0.193^**^ |  |  |  | 0.095 |
| **Positive Mood** |  |  |  | -0.146^*^ |  |  | 0.072 |
| **Worry about COVID-19** |  |  |  |  |  |  |  |
| No worry |  |  |  |  | -0.134 |  | -0.133 |
| Some of time |  |  |  |  | 0.082 |  | 0.076 |
| Most of time |  |  |  |  | -0.112 |  | -0.139 |
| **Loneliness** |  |  |  |  |  | 0.161^*^ | 0.104 |
| *N* | 204 | 204 | 204 | 204 | 204 | 204 | 204 |
| *R*^2^ | 0.108 | 0.086 | 0.107 | 0.092 | 0.109 | 0.095 | 0.158 |
| Adj. *R*^2^ | 0.076 | 0.053 | 0.075 | 0.060 | 0.068 | 0.063 | 0.096 |

**Aggregated Wave 1 and 2 scores**

Supplementary Table: Models predicting belief of having had COVID-19 when asked at wave 3 (Odds Ratios)

|  | (1) | (2) | (3) | (4) | (5) | (6) | (7) |
| --- | --- | --- | --- | --- | --- | --- | --- |
| **Age (per decade)** | 0.883^*^ | 0.851^**^ | 0.837^**^ | 0.826^***^ | 0.822^***^ | 0.836^**^ | 0.899 |
| **Female** | 0.921 | 0.972 | 0.987 | 1.003 | 1.078 | 1.012 | 0.949 |
| **BAME** | 0.761 | 0.741 | 0.699 | 0.706 | 0.738 | 0.708 | 0.733 |
| **Keyworker** | 1.203 | 1.228 | 1.261 | 1.256 | 1.248 | 1.266 | 1.194 |
| **Risk Group^⊥^** |  |  |  |  |  |  |  |
| *At most increased risk* | 0.709 | 0.841 | 0.807 | 0.773 | 1.001 | 0.776 | 0.787 |
| *At increased risk* | 0.957 | 1.000 | 0.996 | 0.996 | 1.072 | 0.975 | 1.016 |
| **Depression** | 1.430^***^ |  |  |  |  |  | 1.577^**^ |
| **Anxiety** |  | 1.222^**^ |  |  |  |  | 0.921 |
| **Stress** |  |  | 1.084^**^ |  |  |  | 1.002 |
| **Positive Mood** |  |  |  | 0.947^**^ |  |  | 0.979 |
| **Worry about COVID-19^¥^** |  |  |  |  | 0.657^*^ |  | 0.540^***^ |
| **Loneliness** |  |  |  |  |  | 1.085^*^ | 0.986 |
| *N* | 878 | 878 | 878 | 878 | 878 | 878 | 878 |
| pseudo *R*^2^ | 0.038 | 0.025 | 0.026 | 0.028 | 0.025 | 0.024 | 0.053 |

^¥^Worry about COVID-19 is treated as a continuous variable in this model, rather than categorical (lower scores indicate less worry, scored 0-3)

Supplementary Table: Models predicting positive COVID-19 test result when asked at wave 3 (Odds Ratios)

|  | (1) | (2) | (3) | (4) | (5) | (6) | (7) |
| --- | --- | --- | --- | --- | --- | --- | --- |
| **Age (per decade)** | 0.795 | 0.831 | 0.814 | 0.795 | 0.791 | 0.803 | 0.744 |
| **Female** | 1.101 | 1.018 | 1.046 | 1.054 | 1.114 | 1.111 | 1.107 |
| **BAME** | 1.263 | 1.279 | 1.164 | 1.143 | 1.258 | 1.260 | 0.985 |
| **Keyworker** | 1.169 | 1.168 | 1.184 | 1.231 | 1.170 | 1.195 | 1.270 |
| **Risk Group^⊥^** |  |  |  |  |  |  |  |
| *At most increased risk* | - | - | - | - | - | - | - |
| *At increased risk* | 0.750 | 0.731 | 0.726 | 0.701 | 0.760 | 0.743 | 0.766 |
| **Depression** | 1.029 |  |  |  |  |  | 0.427^*^ |
| **Anxiety** |  | 1.269 |  |  |  |  | 1.751 |
| **Stress** |  |  | 1.087 |  |  |  | 0.971 |
| **Positive Mood** |  |  |  | 0.919 |  |  | 0.850 |
| **Worry about COVID-19**^¥^ |  |  |  |  | 0.961 |  | 0.706 |
| **Loneliness** |  |  |  |  |  | 1.057 | 0.987 |
| *N* | 370 | 370 | 370 | 370 | 370 | 370 | 370 |
| pseudo *R*^2^ | 0.017 | 0.024 | 0.024 | 0.034 | 0.017 | 0.019 | 0.060 |

^¥^Worry about COVID-19 is treated as a continuous variable in this model, rather than categorical (lower scores indicate less worry, scored 0-3)

Supplementary Table: Models predicting number of COVID-19 symptoms when asked at wave 3 (standardised Betas)

|  | (1) | (2) | (3) | (4) | (5) | (6) | (7) |
| --- | --- | --- | --- | --- | --- | --- | --- |
| **Age (per decade)** | 0.024 | 0.013 | -0.017 | -0.029 | -0.062 | -0.017 | -0.013 |
| **Female** | 0.143^*^ | 0.149^*^ | 0.153^*^ | 0.153^*^ | 0.144^*^ | 0.153^*^ | 0.129 |
| **BAME** | 0.006 | 0.002 | 0.003 | 0.002 | -0.003 | 0.009 | 0.013 |
| **Keyworker** | 0.037 | 0.047 | 0.057 | 0.058 | 0.053 | 0.063 | 0.029 |
| **Risk Group^⊥^** |  |  |  |  |  |  |  |
| *At most increased risk* | -0.007 | 0.003 | 0.001 | 0.004 | -0.003 | 0.003 | -0.016 |
| *At increased risk* | 0.213^**^ | 0.219^**^ | 0.232^**^ | 0.241^***^ | 0.214^**^ | 0.230^**^ | 0.182^*^ |
| **Depression** | 0.221^**^ |  |  |  |  |  | 0.360^**^ |
| **Anxiety** |  | 0.148^*^ |  |  |  |  | -0.106 |
| **Stress** |  |  | 0.082 |  |  |  | -0.022 |
| **Positive Mood** |  |  |  | -0.060 |  |  | 0.132 |
| **Worry about COVID-19^¥^** |  |  |  |  | 0.179^*^ |  | 0.177^*^ |
| **Loneliness** |  |  |  |  |  | 0.073 | 0.016 |
| *N* | 204 | 204 | 204 | 204 | 204 | 204 | 204 |
| *R*^2^ | 0.122 | 0.096 | 0.083 | 0.080 | 0.107 | 0.082 | 0.157 |
| Adj. *R*^2^ | 0.090 | 0.064 | 0.051 | 0.048 | 0.075 | 0.049 | 0.104 |

^¥^Worry about COVID-19 is treated as a continuous variable in this model, rather than categorical (lower scores indicate less worry, scored 0-3)

Supplementary Table: Models predicting COVID-19 symptom severity when asked at wave 3 (standardised Betas)

|  | (1) | (2) | (3) | (4) | (5) | (6) | (7) |
| --- | --- | --- | --- | --- | --- | --- | --- |
| **Age (per decade)** | 0.151^*^ | 0.149^*^ | 0.138 | 0.115 | 0.080 | 0.135 | 0.116 |
| **Female** | -0.006 | -0.002 | 0.005 | 0.006 | -0.005 | 0.004 | -0.003 |
| **BAME** | -0.000 | -0.005 | -0.010 | -0.016 | -0.007 | 0.004 | -0.011 |
| **Keyworker** | 0.126 | 0.133 | 0.141^*^ | 0.143^*^ | 0.142^*^ | 0.154^*^ | 0.136 |
| **Risk Group^⊥^** |  |  |  |  |  |  |  |
| *At most increased risk* | 0.153^*^ | 0.161^*^ | 0.151^*^ | 0.155^*^ | 0.157^*^ | 0.155^*^ | 0.140^*^ |
| *At increased risk* | 0.097 | 0.099 | 0.103 | 0.125 | 0.101 | 0.101 | 0.089 |
| **Depression** | 0.194^**^ |  |  |  |  |  | 0.164 |
| **Anxiety** |  | 0.151^*^ |  |  |  |  | -0.122 |
| **Stress** |  |  | 0.180^*^ |  |  |  | 0.076 |
| **Positive Mood** |  |  |  | -0.179^**^ |  |  | -0.044 |
| **Worry about COVID-19^¥^** |  |  |  |  | 0.135 |  | 0.114 |
| **Loneliness** |  |  |  |  |  | 0.152^*^ | 0.046 |
| *N* | 204 | 204 | 204 | 204 | 204 | 204 | 204 |
| *R*^2^ | 0.106 | 0.091 | 0.102 | 0.102 | 0.088 | 0.093 | 0.126 |
| Adj. *R*^2^ | 0.074 | 0.059 | 0.070 | 0.070 | 0.056 | 0.060 | 0.071 |

^¥^Worry about COVID-19 is treated as a continuous variable in this model, rather than categorical (lower scores indicate less worry, scored 0-3)

**Change from Wave 1 and 2 scores**

Supplementary Table: Models predicting belief of having had COVID-19 when asked at wave 3 (Odds Ratios)

|  | (1) | (2) | (3) | (4) | (5) | (6) | (7) |
| --- | --- | --- | --- | --- | --- | --- | --- |
| **Age (per decade)** | 0.813^***^ | 0.814^***^ | 0.815^***^ | 0.815^***^ | 0.810^***^ | 0.814^***^ | 0.810^***^ |
| **Female** | 1.049 | 1.048 | 1.046 | 1.063 | 1.061 | 1.040 | 1.060 |
| **BAME** | 0.758 | 0.757 | 0.822 | 0.778 | 0.760 | 0.763 | 0.840 |
| **Keyworker** | 1.260 | 1.254 | 1.220 | 1.245 | 1.214 | 1.255 | 1.168 |
| **Risk Group^⊥^** |  |  |  |  |  |  |  |
| *At most increased risk* | 0.883 | 0.885 | 0.881 | 0.885 | 0.951 | 0.893 | 0.962 |
| *At increased risk* | 1.019 | 1.022 | 1.000 | 1.005 | 1.042 | 1.017 | 1.012 |
| **Depression** | 1.054 |  |  |  |  |  | 0.961 |
| **Anxiety** |  | 1.044 |  |  |  |  | 0.956 |
| **Stress** |  |  | 1.080^*^ |  |  |  | 1.088^*^ |
| **Positive Mood** |  |  |  | 0.977 |  |  | 0.995 |
| **Worry about COVID-19**^¥^ |  |  |  |  | 0.785 |  | 0.755^*^ |
| **Loneliness** |  |  |  |  |  | 1.045 | 1.029 |
| *N* | 878 | 878 | 878 | 878 | 878 | 878 | 878 |
| pseudo *R*^2^ | 0.019 | 0.019 | 0.025 | 0.020 | 0.022 | 0.020 | 0.030 |

^¥^Worry about COVID-19 is treated as a continuous variable in this model, rather than categorical (lower scores indicate less worry, scored 0-3)

Supplementary Table: Models predicting positive COVID-19 test result when asked at wave 3 (Odds Ratios)

|  | (1) | (2) | (3) | (4) | (5) | (6) | (7) |
| --- | --- | --- | --- | --- | --- | --- | --- |
| **Age (per decade)** | 0.790 | 0.790 | 0.795 | 0.789 | 0.762 | 0.784 | 0.749 |
| **Female** | 1.083 | 1.107 | 1.094 | 1.130 | 1.273 | 1.129 | 1.453 |
| **BAME** | 1.184 | 1.245 | 1.083 | 1.378 | 1.616 | 1.348 | 1.957 |
| **Keyworker** | 1.176 | 1.172 | 1.167 | 1.175 | 1.053 | 1.174 | 1.068 |
| **Risk Group^⊥^** |  |  |  |  |  |  |  |
| *At most increased risk* | - | - | - | - | - | - | - |
| *At increased risk* | 0.793 | 0.758 | 0.780 | 0.744 | 0.825 | 0.738 | 0.828 |
| **Depression** | 0.844 |  |  |  |  |  | 0.764 |
| **Anxiety** |  | 0.971 |  |  |  |  | 1.220 |
| **Stress** |  |  | 0.928 |  |  |  | 0.893 |
| **Positive Mood** |  |  |  | 0.978 |  |  | 0.936 |
| **Worry about COVID-19**^¥^ |  |  |  |  | 0.438^*^ |  | 0.379^**^ |
| **Loneliness** |  |  |  |  |  | 1.047 | 1.079 |
| *N* | 370 | 370 | 370 | 370 | 370 | 370 | 370 |
| pseudo *R*^2^ | 0.020 | 0.017 | 0.022 | 0.018 | 0.050 | 0.018 | 0.073 |

^¥^Worry about COVID-19 is treated as a continuous variable in this model, rather than categorical (lower scores indicate less worry, scored 0-3)

Supplementary Table: Models predicting number of COVID-19 symptoms when asked at wave 3 (standardised Betas)

|  | (1) | (2) | (3) | (4) | (5) | (6) | (7) |
| --- | --- | --- | --- | --- | --- | --- | --- |
| **Age (per decade)** | -0.034 | -0.033 | -0.033 | -0.035 | -0.039 | -0.035 | -0.033 |
| **Female** | 0.149^*^ | 0.145^*^ | 0.153^*^ | 0.147^*^ | 0.155^*^ | 0.147^*^ | 0.150^*^ |
| **BAME** | 0.003 | 0.000 | 0.016 | 0.004 | 0.010 | 0.004 | 0.021 |
| **Keyworker** | 0.059 | 0.064 | 0.056 | 0.059 | 0.051 | 0.061 | 0.053 |
| **Risk Group^⊥^** |  |  |  |  |  |  |  |
| *At most increased risk* | 0.009 | 0.012 | 0.009 | 0.009 | 0.010 | 0.008 | 0.019 |
| *At increased risk* | 0.241^***^ | 0.242^***^ | 0.238^***^ | 0.240^***^ | 0.243^***^ | 0.241^***^ | 0.240^***^ |
| **Depression** | -0.024 |  |  |  |  |  | 0.023 |
| **Anxiety** |  | -0.083 |  |  |  |  | -0.108 |
| **Stress** |  |  | 0.037 |  |  |  | 0.109 |
| **Positive Mood** |  |  |  | 0.033 |  |  | 0.015 |
| **Worry about COVID-19^¥^** |  |  |  |  | -0.055 |  | -0.045 |
| **Loneliness** |  |  |  |  |  | -0.073 | -0.069 |
| *N* | 204 | 204 | 204 | 204 | 204 | 204 | 204 |
| *R*^2^ | 0.077 | 0.084 | 0.078 | 0.078 | 0.080 | 0.082 | 0.097 |
| Adj. *R*^2^ | 0.045 | 0.051 | 0.045 | 0.045 | 0.047 | 0.050 | 0.040 |

^¥^Worry about COVID-19 is treated as a continuous variable in this model, rather than categorical (lower scores indicate less worry, scored 0-3)

Supplementary Table: Models predicting COVID-19 symptom severity when asked at wave 3 (standardised Betas)

|  | (1) | (2) | (3) | (4) | (5) | (6) | (7) |
| --- | --- | --- | --- | --- | --- | --- | --- |
| **Age (per decade)** | 0.101 | 0.101 | 0.103 | 0.100 | 0.100 | 0.101 | 0.105 |
| **Female** | 0.006 | -0.001 | 0.005 | -0.003 | 0.001 | 0.003 | 0.000 |
| **BAME** | 0.016 | -0.000 | 0.016 | -0.003 | 0.002 | 0.004 | 0.025 |
| **Keyworker** | 0.147^*^ | 0.147^*^ | 0.140^*^ | 0.145^*^ | 0.145^*^ | 0.145^*^ | 0.143^*^ |
| **Risk Group^⊥^** |  |  |  |  |  |  |  |
| *At most increased risk* | 0.161^*^ | 0.166^*^ | 0.168^*^ | 0.167^*^ | 0.166^*^ | 0.165^*^ | 0.171^*^ |
| *At increased risk* | 0.118 | 0.121 | 0.117 | 0.121 | 0.121 | 0.120 | 0.116 |
| **Depression** | 0.070 |  |  |  |  |  | 0.119 |
| **Anxiety** |  | -0.016 |  |  |  |  | -0.091 |
| **Stress** |  |  | 0.071 |  |  |  | 0.093 |
| **Positive Mood** |  |  |  | 0.036 |  |  | 0.114 |
| **Worry about COVID-19^¥^** |  |  |  |  | -0.005 |  | -0.001 |
| **Loneliness** |  |  |  |  |  | 0.045 | 0.048 |
| *N* | 204 | 204 | 204 | 204 | 204 | 204 | 204 |
| *R*^2^ | 0.076 | 0.071 | 0.076 | 0.072 | 0.071 | 0.073 | 0.093 |
| Adj. *R*^2^ | 0.043 | 0.038 | 0.043 | 0.039 | 0.038 | 0.040 | 0.036 |

^¥^Worry about COVID-19 is treated as a continuous variable in this model, rather than categorical (lower scores indicate less worry, scored 0-3)

**Supplementary Appendix S4 – Interaction analyses**

**Composite Measures of Psychological Variables (from PCA analysis)**

**Individual Psychological Predictors**

**Wave 1 Scores**

Supplementary Table: Models predicting belief of having had COVID-19 when asked at wave 3 (Odds Ratios)

|  | (1) | (2) | (3) | (4) | (5) | (6) | (7) |
| --- | --- | --- | --- | --- | --- | --- | --- |
| **Age (per decade)** | 0.869^**^ | 0.849^**^ | 0.826^***^ | 0.823^***^ | 0.828^***^ | 0.830^***^ | 0.885^*^ |
| **Female** | 1.020 | 1.040 | 1.077 | 1.075 | 1.082 | 1.066 | 0.992 |
| **BAME** | 0.770 | 0.765 | 0.754 | 0.737 | 0.764 | 0.773 | 0.805 |
| **Keyworker** | 1.252 | 1.424 | 1.388 | 1.871 | 1.355 | 1.701^*^ | 3.800 |
| **Risk Group^⊥^** |  |  |  |  |  |  |  |
| *At most increased risk* | 0.440 | 0.488 | 0.505 | 0.488 | 0.534 | 0.508 | 0.478 |
| *At increased risk* | 0.885 | 0.911 | 0.927 | 0.928 | 0.938 | 0.919 | 0.925 |
| **Depression** | 1.282^**^ |  |  |  |  |  | 1.293 |
| ***Depression*Keyworker*** | 1.019 |  |  |  |  |  | 1.099 |
| **Anxiety** |  | 1.201^*^ |  |  |  |  | 1.107 |
| ***Anxiety*Keyworker*** |  | 0.971 |  |  |  |  | 0.874 |
| **Stress** |  |  | 1.033 |  |  |  | 0.958 |
| ***Stress*Keyworker*** |  |  | 0.999 |  |  |  | 0.997 |
| **Positive Mood** |  |  |  | 0.975 |  |  | 1.004 |
| ***Positive Mood*Keyworker*** |  |  |  | 0.984 |  |  | 0.964 |
| **Worry about COVID-19** |  |  |  |  |  |  |  |
| No worry |  |  |  |  | 1.738^*^ |  | 1.789^*^ |
| *No worry*keyworker* |  |  |  |  | 0.777 |  | 0.800 |
| Some of time |  |  |  |  | 1.038 |  | 0.853 |
| *Some of time*keyworker* |  |  |  |  | 1.501 |  | 1.469 |
| Most of time |  |  |  |  | 1.568 |  | 0.986 |
| *Most of time*keyworker* |  |  |  |  | 1.246 |  | 1.333 |
| **Loneliness** |  |  |  |  |  | 1.066 | 1.019 |
| ***Loneliness*Keyworker*** |  |  |  |  |  | 0.942 | 0.921 |
| *N* | 1086 | 1086 | 1086 | 1086 | 1086 | 1086 | 1086 |
| *Pseudo R*^2^ | 0.036 | 0.029 | 0.024 | 0.027 | 0.030 | 0.025 | 0.046 |

Supplementary Table: Models predicting having had a positive COVID-19 test when asked at wave 3 (Odds Ratios)

|  | (1) | (2) | (3) | (4) | (5) | (6) | (7) |
| --- | --- | --- | --- | --- | --- | --- | --- |
| **Age (per decade)** | 0.808 | 0.829 | 0.824 | 0.812 | 0.802 | 0.789 | 0.786 |
| **Female** | 1.083 | 1.053 | 1.055 | 1.068 | 0.968 | 1.112 | 1.039 |
| **BAME** | 1.344 | 1.319 | 1.270 | 1.259 | 1.491 | 1.413 | 1.504 |
| **Keyworker** | 0.901 | 1.489 | 1.574 | 1.123 | 1.769 | 1.356 | 0.0598 |
| **Risk Group^⊥^** |  |  |  |  |  |  |  |
| *At most increased risk* | - | - | - | - | - | - | - |
| *At increased risk* | 0.662 | 0.660 | 0.671 | 0.670 | 0.597 | 0.676 | 0.625 |
| **Depression** | 0.962 |  |  |  |  |  | 0.496 |
| ***Depression*Keyworker*** | 1.211 |  |  |  |  |  | 2.305 |
| **Anxiety** |  | 1.136 |  |  |  |  | 1.789 |
| ***Anxiety*Keyworker*** |  | 0.987 |  |  |  |  | 0.516 |
| **Stress** |  |  | 1.052 |  |  |  | 1.044 |
| ***Stress*Keyworker*** |  |  | 0.989 |  |  |  | 1.025 |
| **Positive Mood** |  |  |  | 0.968 |  |  | 0.923 |
| ***Positive Mood*Keyworker*** |  |  |  | 1.014 |  |  | 1.138 |
| **Worry about COVID-19** |  |  |  |  |  |  |  |
| No worry |  |  |  |  | 2.438 |  | 3.558 |
| *No worry*keyworker* |  |  |  |  | 0.270 |  | 0.177 |
| Some of time |  |  |  |  | 2.544 |  | 2.113 |
| *Some of time*keyworker* |  |  |  |  | 0.531 |  | 0.686 |
| Most of time |  |  |  |  | 9.550^**^ |  | 11.92^**^ |
| *Most of time*keyworker* |  |  |  |  | - |  | - |
| **Loneliness** |  |  |  |  |  | 0.931 | 0.873 |
| ***Loneliness*Keyworker*** |  |  |  |  |  | 1.019 | 1.061 |
| *N* | 477 | 477 | 477 | 477 | 470 | 477 | 470 |
| *Pseudo R*^2^ | 0.021 | 0.022 | 0.022 | 0.021 | 0.058 | 0.022 | 0.081 |

Supplementary Table: Models predicting symptom severity when asked at wave 3 (standardised betas)

|  | (1) | (2) | (3) | (4) | (5) | (6) | (7) |
| --- | --- | --- | --- | --- | --- | --- | --- |
| **Age (per decade)** | 0.152^*^ | 0.147^*^ | 0.143^*^ | 0.125^*^ | 0.089 | 0.142^*^ | 0.121 |
| **Female** | 0.004 | 0.014 | 0.016 | 0.021 | 0.011 | 0.016 | 0.007 |
| **BAME** | 0.010 | 0.010 | 0.003 | -0.002 | 0.018 | 0.018 | 0.010 |
| **Keyworker** | 0.122 | 0.198 | 0.260^*^ | -0.013 | 0.100 | 0.141 | 0.029 |
| **Risk Group^⊥^** |  |  |  |  |  |  |  |
| *At most increased risk* | 0.139^*^ | 0.147^*^ | 0.139^*^ | 0.142^*^ | 0.146^*^ | 0.136^*^ | 0.141^*^ |
| *At increased risk* | 0.048 | 0.052 | 0.062 | 0.070 | 0.058 | 0.054 | 0.062 |
| **Depression** | 0.182^*^ |  |  |  |  |  | 0.076 |
| ***Depression*Keyworker*** | -0.014 |  |  |  |  |  | 0.100 |
| **Anxiety** |  | 0.159 |  |  |  |  | -0.103 |
| ***Anxiety*Keyworker*** |  | -0.103 |  |  |  |  | 0.009 |
| **Stress** |  |  | 0.217^**^ |  |  |  | 0.108 |
| ***Stress*Keyworker*** |  |  | -0.163 |  |  |  | -0.175 |
| **Positive Mood** |  |  |  | -0.212^*^ |  |  | -0.093 |
| ***Positive Mood*Keyworker*** |  |  |  | 0.139 |  |  | 0.104 |
| **Worry about COVID-19** |  |  |  |  |  |  |  |
| No worry |  |  |  |  | -0.033 |  | -0.020 |
| *No worry*keyworker* |  |  |  |  | 0.026 |  | 0.006 |
| Some of time |  |  |  |  | 0.035 |  | 0.009 |
| *Some of time*keyworker* |  |  |  |  | 0.004 |  | 0.013 |
| Most of time |  |  |  |  | 0.167 |  | 0.113 |
| *Most of time*keyworker* |  |  |  |  | -0.011 |  | -0.005 |
| **Loneliness** |  |  |  |  |  | 0.161 | 0.054 |
| ***Loneliness*Keyworker*** |  |  |  |  |  | -0.016 | 0.048 |
| *N* | 266 | 266 | 266 | 266 | 266 | 266 | 266 |
| *R*^2^ | 0.080 | 0.065 | 0.078 | 0.083 | 0.077 | 0.073 | 0.110 |
| Adj. *R*^2^ | 0.052 | 0.036 | 0.049 | 0.055 | 0.033 | 0.044 | 0.030 |

Supplementary Table: Models predicting number of symptoms when asked at wave 3 (standardised betas)

|  | (1) | (2) | (3) | (4) | (5) | (6) | (7) |
| --- | --- | --- | --- | --- | --- | --- | --- |
| **Age (per decade)** | 0.040 | 0.021 | 0.003 | -0.004 | -0.038 | 0.004 | 0.001 |
| **Female** | 0.121^*^ | 0.136^*^ | 0.142^*^ | 0.146^*^ | 0.133^*^ | 0.141^*^ | 0.113 |
| **BAME** | -0.005 | -0.007 | -0.005 | -0.005 | -0.001 | 0.002 | 0.014 |
| **Keyworker** | -0.044 | 0.014 | 0.094 | -0.165 | 0.048 | 0.027 | -0.735 |
| **Risk Group^⊥^** |  |  |  |  |  |  |  |
| *At most increased risk* | 0.001 | 0.007 | 0.006 | 0.011 | 0.006 | 0.003 | -0.002 |
| *At increased risk* | 0.162^**^ | 0.167^**^ | 0.180^**^ | 0.185^**^ | 0.175^**^ | 0.175^**^ | 0.146^*^ |
| **Depression** | 0.212^**^ |  |  |  |  |  | 0.404^**^ |
| ***Depression*Keyworker*** | 0.068 |  |  |  |  |  | 0.028 |
| **Anxiety** |  | 0.110 |  |  |  |  | -0.195 |
| ***Anxiety*Keyworker*** |  | 0.012 |  |  |  |  | 0.119 |
| **Stress** |  |  | 0.092 |  |  |  | -0.081 |
| ***Stress*Keyworker*** |  |  | -0.077 |  |  |  | 0.118 |
| **Positive Mood** |  |  |  | -0.112 |  |  | 0.019 |
| ***Positive Mood*Keyworker*** |  |  |  | 0.205 |  |  | 0.535 |
| **Worry about COVID-19** |  |  |  |  |  |  |  |
| No worry |  |  |  |  | -0.020 |  | -0.072 |
| *No worry*keyworker* |  |  |  |  | -0.096 |  | -0.077 |
| Some of time |  |  |  |  | 0.047 |  | 0.037 |
| *Some of time*keyworker* |  |  |  |  | -0.037 |  | --0.061 |
| Most of time |  |  |  |  | 0.070 |  | 0.011 |
| *Most of time*keyworker* |  |  |  |  | 0.094 |  | 0.130 |
| **Loneliness** |  |  |  |  |  | 0.075 | 0.015 |
| ***Loneliness*Keyworker*** |  |  |  |  |  | 0.008 | 0.054 |
| *N* | 266 | 266 | 266 | 266 | 266 | 266 | 266 |
| *R*^2^ | 0.102 | 0.063 | 0.056 | 0.058 | 0.089 | 0.057 | 0.160 |
| Adj. *R*^2^ | 0.074 | 0.034 | 0.026 | 0.028 | 0.046 | 0.028 | 0.084 |

**Wave 2 Scores**

Supplementary Table: Models predicting belief of having had COVID-19 when asked at wave 3 (Odds Ratios)

|  | (1) | (2) | (3) | (4) | (5) | (6) | (7) |
| --- | --- | --- | --- | --- | --- | --- | --- |
| **Age (per decade)** | 0.861^*^ | 0.839^**^ | 0.837^**^ | 0.825^***^ | 0.817^***^ | 0.836^**^ | 0.861^*^ |
| **Female** | 0.947 | 0.988 | 0.982 | 1.021 | 1.097 | 1.001 | 1.011 |
| **BAME** | 0.787 | 0.755 | 0.741 | 0.744 | 0.766 | 0.716 | 0.779 |
| **Keyworker** | 0.918 | 0.967 | 1.028 | 1.664 | 1.153 | 1.359 | 0.614 |
| **Risk Group^⊥^** |  |  |  |  |  |  |  |
| *At most increased risk* | 0.738 | 0.839 | 0.793 | 0.767 | 1.188 | 0.774 | 0.991 |
| *At increased risk* | 0.952 | 0.996 | 0.979 | 0.975 | 1.095 | 0.967 | 1.019 |
| **Depression** | 1.287^*^ |  |  |  |  |  | 1.310 |
| ***Depression*Keyworker*** | 1.140 |  |  |  |  |  | 1.079 |
| **Anxiety** |  | 1.132 |  |  |  |  | 0.860 |
| ***Anxiety*Keyworker*** |  | 1.136 |  |  |  |  | 1.112 |
| **Stress** |  |  | 1.082^*^ |  |  |  | 1.039 |
| ***Stress*Keyworker*** |  |  | 1.033 |  |  |  | 1.037 |
| **Positive Mood** |  |  |  | 0.953^*^ |  |  | 0.977 |
| ***Positive Mood*Keyworker*** |  |  |  | 0.986 |  |  | 1.013 |
| **Worry about COVID-19** |  |  |  |  |  |  |  |
| No worry |  |  |  |  | 1.636 |  | 1.766^*^ |
| *No worry*keyworker* |  |  |  |  | 1.091 |  | 1.144 |
| Some of time |  |  |  |  | 0.670 |  | 0.510 |
| *Some of time*keyworker* |  |  |  |  | 0.966 |  | 0.927 |
| Most of time |  |  |  |  | 3.136 |  | 3.336 |
| *Most of time*keyworker* |  |  |  |  | - |  | - |
| **Loneliness** |  |  |  |  |  | 1.099^*^ | 1.037 |
| ***Loneliness*Keyworker*** |  |  |  |  |  | 0.980 | 0.927 |
| *N* | 878 | 878 | 878 | 878 | 869 | 878 | 869 |
| *Pseudo R*^2^ | 0.037 | 0.026 | 0.032 | 0.029 | 0.029 | 0.026 | 0.060 |

Supplementary Table: Models predicting having had a positive COVID-19 test when asked at wave 3 (Odds Ratios)

|  | (1) | (2) | (3) | (4) | (5) | (6) | (7) |
| --- | --- | --- | --- | --- | --- | --- | --- |
| **Age (per decade)** | 0.795 | 0.851 | 0.817 | 0.803 | 0.790 | 0.807 | 0.741 |
| **Female** | 1.128 | 1.024 | 1.075 | 1.138 | 1.179 | 1.127 | 1.239 |
| **BAME** | 1.212 | 1.296 | 1.146 | 1.240 | 1.216 | 1.343 | 1.232 |
| **Keyworker** | 1.856 | 2.706 | 3.493 | 0.167 | 0.930 | 2.455 | 1.306 |
| **Risk Group^⊥^** |  |  |  |  |  |  |  |
| *At most increased risk* | - | - | - | - | - | - | - |
| *At increased risk* | 0.746 | 0.720 | 0.712 | 0.662 | 0.899 | 0.773 | 1.194 |
| **Depression** | 1.074 |  |  |  |  |  | 0.303^*^ |
| ***Depression*Keyworker*** | 0.803 |  |  |  |  |  | 2.430 |
| **Anxiety** |  | 1.482 |  |  |  |  | 2.054 |
| ***Anxiety*Keyworker*** |  | 0.663 |  |  |  |  | 0.670 |
| **Stress** |  |  | 1.134 |  |  |  | 1.060 |
| ***Stress*Keyworker*** |  |  | 0.831 |  |  |  | 0.843 |
| **Positive Mood** |  |  |  | 0.888^*^ |  |  | 0.851 |
| ***Positive Mood*Keyworker*** |  |  |  | 1.108 |  |  | 1.026 |
| **Worry about COVID-19** |  |  |  |  |  |  |  |
| No worry |  |  |  |  | 0.804 |  | 1.138 |
| *No worry*keyworker* |  |  |  |  | 2.356 |  | 1.855 |
| Some of time |  |  |  |  | - |  | - |
| *Some of time*keyworker* |  |  |  |  | - |  | - |
| Most of time |  |  |  |  | - |  | - |
| *Most of time*keyworker* |  |  |  |  | - |  | - |
| **Loneliness** |  |  |  |  |  | 1.176 | 1.086 |
| ***Loneliness*Keyworker*** |  |  |  |  |  | 0.809 | 0.818 |
| *N* | 370 | 370 | 370 | 370 | 345 | 370 | 345 |
| *Pseudo R*^2^ | 0.019 | 0.029 | 0.029 | 0.041 | 0.025 | 0.029 | 0.097 |

Supplementary Table: Models predicting symptom severity when asked at wave 3 (standardised betas)

|  | (1) | (2) | (3) | (4) | (5) | (6) | (7) |
| --- | --- | --- | --- | --- | --- | --- | --- |
| **Age (per decade)** | 0.158^*^ | 0.144 | 0.142^*^ | 0.110 | 0.098 | 0.137 | 0.148 |
| **Female** | 0.005 | 0.003 | 0.014 | 0.016 | -0.009 | 0.009 | 0.006 |
| **BAME** | 0.015 | 0.004 | 0.005 | -0.009 | -0.006 | 0.007 | 0.014 |
| **Keyworker** | 0.286 | 0.254 | 0.310^*^ | -0.142 | 0.156 | 0.209 | -0.342 |
| **Risk Group^⊥^** |  |  |  |  |  |  |  |
| *At most increased risk* | 0.148^*^ | 0.163^*^ | 0.166^*^ | 0.163^*^ | 0.153^*^ | 0.158^*^ | 0.149^*^ |
| *At increased risk* | 0.101 | 0.110 | 0.108 | 0.131 | 0.125 | 0.106 | 0.120 |
| **Depression** | 0.273^**^ |  |  |  |  |  | 0.274 |
| ***Depression*Keyworker*** | -0.193 |  |  |  |  |  | -0.081 |
| **Anxiety** |  | 0.193^*^ |  |  |  |  | -0.146 |
| ***Anxiety*Keyworker*** |  | -0.159 |  |  |  |  | -0.073 |
| **Stress** |  |  | 0.273^**^ |  |  |  | 0.154 |
| ***Stress*Keyworker*** |  |  | -0.215 |  |  |  | -0.154 |
| **Positive Mood** |  |  |  | -0.206^*^ |  |  | -0.043 |
| ***Positive Mood*Keyworker*** |  |  |  | 0.300 |  |  | 0.619 |
| **Worry about COVID-19** |  |  |  |  |  |  |  |
| No worry |  |  |  |  | -0.128 |  | -0.145 |
| *No worry*keyworker* |  |  |  |  | -0.003 |  | -0.024 |
| Some of time |  |  |  |  | 0.020 |  | -0.012 |
| *Some of time*keyworker* |  |  |  |  | 0.089 |  | 0.146 |
| Most of time |  |  |  |  | -0.113 |  | 0.187^*^ |
| *Most of time*keyworker* |  |  |  |  | - |  | - |
| **Loneliness** |  |  |  |  |  | 0.200^*^ | 0.004 |
| ***Loneliness*Keyworker*** |  |  |  |  |  | -0.080 | 0.229 |
| *N* | 204 | 204 | 204 | 204 | 204 | 204 | 204 |
| *R*^2^ | 0.113 | 0.091 | 0.114 | 0.097 | 0.113 | 0.097 | 0.191 |
| Adj. *R*^2^ | 0.076 | 0.054 | 0.078 | 0.060 | 0.062 | 0.060 | 0.098 |

Supplementary Table: Models predicting number of symptoms when asked at wave 3 (standardised betas)

|  | (1) | (2) | (3) | (4) | (5) | (6) | (7) |
| --- | --- | --- | --- | --- | --- | --- | --- |
| **Age (per decade)** | 0.023 | -0.002 | -0.014 | -0.032 | -0.035 | -0.023 | 0.008 |
| **Female** | 0.156^*^ | 0.153^*^ | 0.158^*^ | 0.156^*^ | 0.139^*^ | 0.154^*^ | 0.138* |
| **BAME** | 0.019 | 0.010 | 0.010 | 0.004 | -0.002 | 0.007 | 0.013 |
| **Keyworker** | 0.240 | 0.119 | 0.184 | -0.081 | 0.129 | 0.198 | 0.150 |
| **Risk Group^⊥^** |  |  |  |  |  |  |  |
| *At most increased risk* | -0.009 | 0.005 | 0.011 | 0.008 | -0.006 | 0.012 | -0.021 |
| *At increased risk* | 0.223^**^ | 0.230^**^ | 0.236^**^ | 0.245^***^ | 0.236^**^ | 0.249^***^ | 0.225^**^ |
| **Depression** | 0.274^**^ |  |  |  |  |  | 0.157 |
| ***Depression*Keyworker*** | -0.241 |  |  |  |  |  | 0.388 |
| **Anxiety** |  | 0.135 |  |  |  |  | -0.071 |
| ***Anxiety*Keyworker*** |  | -0.092 |  |  |  |  | -0.121 |
| **Stress** |  |  | 0.150 |  |  |  | 0.065 |
| ***Stress*Keyworker*** |  |  | -0.159 |  |  |  | -0.048 |
| **Positive Mood** |  |  |  | -0.069 |  |  | 0.206 |
| ***Positive Mood*Keyworker*** |  |  |  | 0.146 |  |  | -0.228 |
| **Worry about COVID-19** |  |  |  |  |  |  |  |
| No worry |  |  |  |  | -0.035 |  | -0.268 |
| *No worry*keyworker* |  |  |  |  | -0.162 |  | -0.137 |
| Some of time |  |  |  |  | -0.022 |  | 0.125 |
| *Some of time*keyworker* |  |  |  |  | 0.100 |  | -0.114 |
| Most of time |  |  |  |  | -0.035 |  | -0.063 |
| *Most of time*keyworker* |  |  |  |  | - |  | 0- |
| **Loneliness** |  |  |  |  |  | 0.130 | -0.053 |
| ***Loneliness*Keyworker*** |  |  |  |  |  | -0.193 | 0.77 |
| *N* | 204 | 204 | 204 | 204 | 204 | 204 | 204 |
| *R*^2^ | 0.115 | 0.087 | 0.089 | 0.080 | 0.117 | 0.088 | 0.176 |
| Adj. *R*^2^ | 0.079 | 0.050 | 0.052 | 0.042 | 0.066 | 0.051 | 0.081 |

**Aggregated Wave 1 and Wave 2 Scores**

Supplementary Table: Models predicting belief of having had COVID-19 when asked at wave 3 (Odds Ratios)

|  | (1) | (2) | (3) | (4) | (5) | (6) | (7) |
| --- | --- | --- | --- | --- | --- | --- | --- |
| **Age (per decade)** | 0.877^*^ | 0.848^**^ | 0.834^**^ | 0.825^***^ | 0.822^***^ | 0.838^**^ | 0.892 |
| **Female** | 0.925 | 0.976 | 0.993 | 1.002 | 1.082 | 1.003 | 0.940 |
| **BAME** | 0.760 | 0.740 | 0.702 | 0.711 | 0.744 | 0.711 | 0.744 |
| **Keyworker** | 0.895 | 1.078 | 1.022 | 2.030 | 0.789 | 1.469 | 1.148 |
| **Risk Group^⊥^** |  |  |  |  |  |  |  |
| *At most increased risk* | 0.720 | 0.843 | 0.802 | 0.767 | 1.021 | 0.777 | 0.817 |
| *At increased risk* | 0.953 | 0.995 | 0.996 | 0.992 | 1.074 | 0.980 | 1.032 |
| **Depression** | 1.360^**^ |  |  |  |  |  | 1.413 |
| ***Depression*Keyworker*** | 1.134 |  |  |  |  |  | 1.272 |
| **Anxiety** |  | 1.189 |  |  |  |  | 0.952 |
| ***Anxiety*Keyworker*** |  | 1.066 |  |  |  |  | 0.917 |
| **Stress** |  |  | 1.068 |  |  |  | 0.991 |
| ***Stress*Keyworker*** |  |  | 1.037 |  |  |  | 1.021 |
| **Positive Mood** |  |  |  | 0.955^*^ |  |  | 0.984 |
| ***Positive Mood*Keyworker*** |  |  |  | 0.976 |  |  | 0.979 |
| **Worry about COVID-19^¥^** |  |  |  |  | 0.582^*^ |  | 0.481^**^ |
| ***Worry about COVID-19*Keyworker*** |  |  |  |  | 1.279 |  | 1.214 |
| **Loneliness** |  |  |  |  |  | 1.106^*^ | 1.043 |
| ***Loneliness*Keyworker*** |  |  |  |  |  | 0.958 | 0.888 |
| *N* | 878 | 878 | 878 | 878 | 878 | 878 | 878 |
| *Pseudo R*^2^ | 0.038 | 0.026 | 0.027 | 0.028 | 0.026 | 0.024 | 0.056 |

Worry about COVID-19 is treated as a continuous variable in this model, rather than categorical (lower scores indicate less worry, scored 0-3)

Supplementary Table: Models predicting having had a positive COVID-19 test when asked at wave 3 (Odds Ratios)

|  | (1) | (2) | (3) | (4) | (5) | (6) | (7) |
| --- | --- | --- | --- | --- | --- | --- | --- |
| **Age (per decade)** | 0.802 | 0.851 | 0.831 | 0.807 | 0.791 | 0.805 | 0.729 |
| **Female** | 1.100 | 0.994 | 1.032 | 1.082 | 1.115 | 1.099 | 1.151 |
| **BAME** | 1.247 | 1.264 | 1.120 | 1.075 | 1.270 | 1.297 | 1.116 |
| **Keyworker** | 1.551 | 2.106 | 2.994 | 0.135 | 0.994 | 1.717 | 0.001 |
| **Risk Group^⊥^** |  |  |  |  |  |  |  |
| *At most increased risk* | - | - | - | - | - | - | - |
| *At increased risk* | 0.745 | 0.738 | 0.717 | 0.666 | 0.761 | 0.762 | 0.852 |
| **Depression** | 1.095 |  |  |  |  |  | 0.123^*^ |
| ***Depression*Keyworker*** | 0.884 |  |  |  |  |  | 5.966 |
| **Anxiety** |  | 1.455 |  |  |  |  | 3.625^*^ |
| ***Anxiety*Keyworker*** |  | 0.764 |  |  |  |  | 0.385 |
| **Stress** |  |  | 1.161 |  |  |  | 1.012 |
| ***Stress*Keyworker*** |  |  | 0.863 |  |  |  | 0.923 |
| **Positive Mood** |  |  |  | 0.876^*^ |  |  | 0.741^*^ |
| ***Positive Mood*Keyworker*** |  |  |  | 1.122 |  |  | 1.276 |
| **Worry about COVID-19^¥^** |  |  |  |  | 0.916 |  | 0.419 |
| ***Worry about COVID-19*Keyworker*** |  |  |  |  | 1.091 |  | 2.193 |
| **Loneliness** |  |  |  |  |  | 1.107 | 1.035 |
| ***Loneliness*Keyworker*** |  |  |  |  |  | 0.902 | 0.937 |
| *N* | 370 | 370 | 370 | 370 | 370 | 370 | 370 |
| *Pseudo R*^2^ | 0.017 | 0.027 | 0.029 | 0.041 | 0.017 | 0.020 | 0.091 |

Worry about COVID-19 is treated as a continuous variable in this model, rather than categorical (lower scores indicate less worry, scored 0-3)

Supplementary Table: Models predicting symptom severity when asked at wave 3 (standardised betas)

|  | (1) | (2) | (3) | (4) | (5) | (6) | (7) |
| --- | --- | --- | --- | --- | --- | --- | --- |
| **Age (per decade)** | 0.155^*^ | 0.153^*^ | 0.141^*^ | 0.115 | 0.080 | 0.136 | 0.117 |
| **Female** | -0.005 | -0.001 | 0.009 | 0.012 | -0.005 | 0.004 | 0.007 |
| **BAME** | -0.000 | -0.003 | -0.010 | -0.018 | -0.007 | 0.004 | -0.019 |
| **Keyworker** | 0.199 | 0.210 | 0.365^*^ | -0.205 | 0.106 | 0.180 | -0.060 |
| **Risk Group^⊥^** |  |  |  |  |  |  |  |
| *At most increased risk* | 0.154^*^ | 0.165^*^ | 0.166^*^ | 0.166^*^ | 0.158^*^ | 0.157^*^ | 0.158^*^ |
| *At increased risk* | 0.100 | 0.104 | 0.113 | 0.133 | 0.102 | 0.104 | 0.095 |
| **Depression** | 0.220^*^ |  |  |  |  |  | 0.164 |
| ***Depression*Keyworker*** | -0.084 |  |  |  |  |  | -0.012 |
| **Anxiety** |  | 0.188 |  |  |  |  | -0.166 |
| ***Anxiety*Keyworker*** |  | -0.095 |  |  |  |  | 0.138 |
| **Stress** |  |  | 0.273^**^ |  |  |  | 0.197 |
| ***Stress*Keyworker*** |  |  | -0.265 |  |  |  | -0.359 |
| **Positive Mood** |  |  |  | -0.249^**^ |  |  | -0.109 |
| ***Positive Mood*Keyworker*** |  |  |  | 0.362 |  |  | 0.273 |
| **Worry about COVID-19^¥^** |  |  |  |  | 0.124 |  | 0.099 |
| ***Worry about COVID-19*Keyworker*** |  |  |  |  | 0.038 |  | 0.023 |
| **Loneliness** |  |  |  |  |  | 0.168 | -0.030 |
| ***Loneliness*Keyworker*** |  |  |  |  |  | -0.035 | 0.152 |
| *N* | 204 | 204 | 204 | 204 | 204 | 204 | 204 |
| *R*^2^ | 0.106 | 0.093 | 0.112 | 0.108 | 0.089 | 0.093 | 0.141 |
| Adj. *R*^2^ | 0.070 | 0.056 | 0.076 | 0.072 | 0.051 | 0.056 | 0.057 |

Worry about COVID-19 is treated as a continuous variable in this model, rather than categorical (lower scores indicate less worry, scored 0-3)

Supplementary Table: Models predicting number of symptoms when asked at wave 3 (standardised betas)

|  | (1) | (2) | (3) | (4) | (5) | (6) | (7) |
| --- | --- | --- | --- | --- | --- | --- | --- |
| **Age (per decade)** | 0.028 | 0.012 | -0.015 | -0.029 | -0.056 | -0.016 | -0.013 |
| **Female** | 0.145^*^ | 0.148^*^ | 0.155^*^ | 0.157^*^ | 0.143^*^ | 0.151^*^ | 0.143^*^ |
| **BAME** | 0.006 | 0.002 | 0.003 | 0.001 | -0.001 | 0.011 | 0.001 |
| **Keyworker** | 0.127 | 0.024 | 0.182 | -0.213 | -0.343 | 0.131 | -0.731 |
| **Risk Group^⊥^** |  |  |  |  |  |  |  |
| *At most increased risk* | -0.005 | 0.002 | 0.009 | 0.012 | -0.001 | 0.006 | -0.014 |
| *At increased risk* | 0.216^**^ | 0.218^**^ | 0.238^***^ | 0.248^***^ | 0.224^**^ | 0.237^**^ | 0.190^**^ |
| **Depression** | 0.253^**^ |  |  |  |  |  | 0.528^**^ |
| ***Depression*Keyworker*** | -0.104 |  |  |  |  |  | -0.518 |
| **Anxiety** |  | 0.137 |  |  |  |  | -0.316 |
| ***Anxiety*Keyworker*** |  | 0.028 |  |  |  |  | 0.431 |
| **Stress** |  |  | 0.134 |  |  |  | -0.003 |
| ***Stress*Keyworker*** |  |  | -0.148 |  |  |  | 0.067 |
| **Positive Mood** |  |  |  | -0.114 |  |  | 0.042 |
| ***Positive Mood*Keyworker*** |  |  |  | 0.286 |  |  | 0.548 |
| **Worry about COVID-19^¥^** |  |  |  |  | 0.056 |  | 0.125 |
| ***Worry about COVID-19*Keyworker*** |  |  |  |  | 0.433 |  | 0.286 |
| **Loneliness** |  |  |  |  |  | 0.113 | 0.000 |
| ***Loneliness*Keyworker*** |  |  |  |  |  | -0.090 | 0.027 |
| *N* | 204 | 204 | 204 | 204 | 204 | 204 | 204 |
| *R*^2^ | 0.123 | 0.097 | 0.087 | 0.084 | 0.120 | 0.084 | 0.182 |
| Adj. *R*^2^ | 0.087 | 0.059 | 0.049 | 0.047 | 0.084 | 0.046 | 0.103 |

Worry about COVID-19 is treated as a continuous variable in this model, rather than categorical (lower scores indicate less worry, scored 0-3)

**Change from Wave 1 to Wave 2 Scores**

Supplementary Table: Models predicting belief of having had COVID-19 when asked at wave 3 (Odds Ratios)

|  | (1) | (2) | (3) | (4) | (5) | (6) | (7) |
| --- | --- | --- | --- | --- | --- | --- | --- |
| **Age (per decade)** | 0.815^***^ | 0.814^***^ | 0.815^***^ | 0.813^***^ | 0.810^***^ | 0.814^***^ | 0.804^***^ |
| **Female** | 1.048 | 1.043 | 1.045 | 1.060 | 1.055 | 1.034 | 1.028 |
| **BAME** | 0.770 | 0.769 | 0.823 | 0.767 | 0.767 | 0.772 | 0.862 |
| **Keyworker** | 1.297 | 1.321 | 1.221 | 1.220 | 1.177 | 1.263 | 1.182 |
| **Risk Group^⊥^** |  |  |  |  |  |  |  |
| *At most increased risk* | 0.896 | 0.877 | 0.883 | 0.870 | 0.946 | 0.897 | 0.912 |
| *At increased risk* | 1.032 | 1.050 | 1.000 | 1.004 | 1.042 | 1.021 | 1.064 |
| **Depression** | 0.975 |  |  |  |  |  | 0.901 |
| ***Depression*Keyworker*** | 1.181 |  |  |  |  |  | 1.172 |
| **Anxiety** |  | 0.932 |  |  |  |  | 0.816 |
| ***Anxiety*Keyworker*** |  | 1.270 |  |  |  |  | 1.392 |
| **Stress** |  |  | 1.078 |  |  |  | 1.098 |
| ***Stress*Keyworker*** |  |  | 1.005 |  |  |  | 0.990 |
| **Positive Mood** |  |  |  | 0.964 |  |  | 0.959 |
| ***Positive Mood*Keyworker*** |  |  |  | 1.031 |  |  | 1.096 |
| **Worry about COVID-19^¥^** |  |  |  |  | 0.850 |  | 0.823 |
| ***Worry about COVID-19*Keyworker*** |  |  |  |  | 0.839 |  | 0.816 |
| **Loneliness** |  |  |  |  |  | 1.029 | 1.007 |
| ***Loneliness*Keyworker*** |  |  |  |  |  | 1.030 | 1.045 |
| *N* | 878 | 878 | 878 | 878 | 878 | 878 | 878 |
| *Pseudo R*^2^ | 0.020 | 0.021 | 0.025 | 0.020 | 0.022 | 0.020 | 0.037 |

Worry about COVID-19 is treated as a continuous variable in this model, rather than categorical (lower scores indicate less worry, scored 0-3)

Supplementary Table: Models predicting having had a positive COVID-19 test when asked at wave 3 (Odds Ratios)

|  | (1) | (2) | (3) | (4) | (5) | (6) | (7) |
| --- | --- | --- | --- | --- | --- | --- | --- |
| **Age (per decade)** | 0.786 | 0.791 | 0.791 | 0.787 | 0.757 | 0.781 | 0.729 |
| **Female** | 1.113 | 1.141 | 1.101 | 1.134 | 1.142 | 1.227 | 1.364 |
| **BAME** | 1.150 | 1.192 | 1.027 | 1.362 | 1.725 | 1.186 | 1.816 |
| **Keyworker** | 1.037 | 1.021 | 1.069 | 1.152 | 0.703 | 1.133 | 0.563 |
| **Risk Group^⊥^** |  |  |  |  |  |  |  |
| *At most increased risk* | - | - | - | - | - | - | - |
| *At increased risk* | 0.765 | 0.713 | 0.756 | 0.739 | 0.794 | 0.736 | 0.812 |
| **Depression** | 0.992 |  |  |  |  |  | 0.833 |
| ***Depression*Keyworker*** | 0.738 |  |  |  |  |  | 0.859 |
| **Anxiety** |  | 1.273 |  |  |  |  | 1.345 |
| ***Anxiety*Keyworker*** |  | 0.601 |  |  |  |  | 0.853 |
| **Stress** |  |  | 0.986 |  |  |  | 0.922 |
| ***Stress*Keyworker*** |  |  | 0.901 |  |  |  | 0.886 |
| **Positive Mood** |  |  |  | 0.963 |  |  | 0.987 |
| ***Positive Mood*Keyworker*** |  |  |  | 1.031 |  |  | 0.901 |
| **Worry about COVID-19^¥^** |  |  |  |  | 0.876 |  | 0.786 |
| ***Worry about COVID-19*Keyworker*** |  |  |  |  | 0.281 |  | 0.244 |
| **Loneliness** |  |  |  |  |  | 1.302 | 1.304 |
| ***Loneliness*Keyworker*** |  |  |  |  |  | 0.711 | 0.763 |
| *N* | 370 | 370 | 370 | 370 | 370 | 370 | 370 |
| *Pseudo R*^2^ | 0.023 | 0.024 | 0.025 | 0.018 | 0.070 | 0.035 | 0.116 |

Worry about COVID-19 is treated as a continuous variable in this model, rather than categorical (lower scores indicate less worry, scored 0-3)

Supplementary Table: Models predicting symptom severity when asked at wave 3 (standardised betas)

|  | (1) | (2) | (3) | (4) | (5) | (6) | (7) |
| --- | --- | --- | --- | --- | --- | --- | --- |
| **Age (per decade)** | 0.103 | 0.107 | 0.103 | 0.100 | 0.099 | 0.102 | 0.118 |
| **Female** | 0.006 | -0.000 | 0.004 | -0.003 | 0.001 | 0.007 | -0.002 |
| **BAME** | 0.010 | -0.003 | 0.016 | -0.004 | 0.001 | -0.002 | 0.016 |
| **Keyworker** | 0.137 | 0.127 | 0.140^*^ | 0.144^*^ | 0.136 | 0.141^*^ | 0.106 |
| **Risk Group^⊥^** |  |  |  |  |  |  |  |
| *At most increased risk* | 0.154^*^ | 0.156^*^ | 0.168^*^ | 0.167^*^ | 0.166^*^ | 0.165^*^ | 0.150^*^ |
| *At increased risk* | 0.116 | 0.120 | 0.117 | 0.121 | 0.124 | 0.120 | 0.111 |
| **Depression** | 0.129 |  |  |  |  |  | 0.190 |
| ***Depression*Keyworker*** | -0.086 |  |  |  |  |  | -0.094 |
| **Anxiety** |  | 0.069 |  |  |  |  | 0.014 |
| ***Anxiety*Keyworker*** |  | -0.130 |  |  |  |  | -0.149 |
| **Stress** |  |  | 0.060 |  |  |  | 0.002 |
| ***Stress*Keyworker*** |  |  | -0.016 |  |  |  | -0.112 |
| **Positive Mood** |  |  |  | 0.030 |  |  | 0.168 |
| ***Positive Mood*Keyworker*** |  |  |  | 0.009 |  |  | -0.064 |
| **Worry about COVID-19^¥^** |  |  |  |  | 0.031 |  | 0.046 |
| ***Worry about COVID-19*Keyworker*** |  |  |  |  | -0.050 |  | -0.042 |
| **Loneliness** |  |  |  |  |  | 0.095 | 0.083 |
| ***Loneliness*Keyworker*** |  |  |  |  |  | -0.069 | -0.030 |
| *N* | 204 | 204 | 204 | 204 | 204 | 204 | 204 |
| *R*^2^ | 0.079 | 0.081 | 0.076 | 0.072 | 0.072 | 0.075 | 0.110 |
| Adj. *R*^2^ | 0.042 | 0.043 | 0.038 | 0.034 | 0.034 | 0.037 | 0.024 |

Worry about COVID-19 is treated as a continuous variable in this model, rather than categorical (lower scores indicate less worry, scored 0-3)

Supplementary Table: Models predicting number of symptoms when asked at wave 3 (standardised betas)

|  | (1) | (2) | (3) | (4) | (5) | (6) | (7) |
| --- | --- | --- | --- | --- | --- | --- | --- |
| **Age (per decade)** | -0.032 | -0.024 | -0.033 | -0.032 | -0.039 | -0.032 | -0.006 |
| **Female** | 0.149^*^ | 0.146^*^ | 0.154^*^ | 0.147^*^ | 0.155^*^ | 0.158^*^ | 0.157^*^ |
| **BAME** | -0.006 | -0.004 | 0.015 | 0.010 | 0.010 | -0.012 | 0.015 |
| **Keyworker** | 0.045 | 0.036 | 0.057 | 0.065 | 0.051 | 0.052 | 0.039 |
| **Risk Group^⊥^** |  |  |  |  |  |  |  |
| *At most increased risk* | -0.001 | -0.003 | 0.009 | 0.011 | 0.010 | 0.007 | -0.004 |
| *At increased risk* | 0.237^***^ | 0.241^***^ | 0.238^***^ | 0.238^***^ | 0.243^***^ | 0.241^***^ | 0.223^**^ |
| **Depression** | 0.062 |  |  |  |  |  | 0.105 |
| ***Depression*Keyworker*** | -0.124 |  |  |  |  |  | -0.095 |
| **Anxiety** |  | 0.040 |  |  |  |  | 0.029 |
| ***Anxiety*Keyworker*** |  | -0.188 |  |  |  |  | -0.198 |
| **Stress** |  |  | 0.068 |  |  |  | 0.075 |
| ***Stress*Keyworker*** |  |  | -0.044 |  |  |  | 0.017 |
| **Positive Mood** |  |  |  | 0.079 |  |  | 0.198 |
| ***Positive Mood*Keyworker*** |  |  |  | -0.067 |  |  | -0.263 |
| **Worry about COVID-19^¥^** |  |  |  |  | -0.053 |  | -0.041 |
| ***Worry about COVID-19*Keyworker*** |  |  |  |  | -0.002 |  | 0.029 |
| **Loneliness** |  |  |  |  |  | 0.056 | 0.043 |
| ***Loneliness*Keyworker*** |  |  |  |  |  | -0.177 | -0.159 |
| *N* | 204 | 204 | 204 | 204 | 204 | 204 | 204 |
| *R*^2^ | 0.085 | 0.103 | 0.079 | 0.080 | 0.080 | 0.096 | 0.142 |
| Adj. *R*^2^ | 0.048 | 0.066 | 0.041 | 0.043 | 0.042 | 0.059 | 0.058 |

Worry about COVID-19 is treated as a continuous variable in this model, rather than categorical (lower scores indicate less worry, scored 0-3)
